# Cilastatin Protects Against Chemotherapy-Induced Neuropathy via the FKBP51/DPEP1 Axis: Confocal Insights into Protein Dynamics

**DOI:** 10.3390/cells15191737

**Published:** 2026-09-23

**Authors:** Rita Martín-Ramírez, Karen Álvarez-Tosco, María Ángeles González-Nicolás, Elena Vázquez-Ogando, Alberto Lázaro, Felipe-Manuel Rosa-González, Julio Ávila, Manuel Morales, Pablo Martín-Vasallo

**Affiliations:** 1 Laboratorio de Biología del Desarrollo, UD de Bioquímica y Biología Molecular, Universidad de La Laguna, 38206 San Cristóbal de La Laguna, Spain; rmartira@ull.edu.es (R.M.-R.); karenalvtos@gmail.com (K.Á.-T.); javila@ull.edu.es (J.Á.); 2 Instituto de Tecnologías Biomédicas, Universidad de La Laguna, 38071 San Cristóbal de La Laguna, Spain; 3 Departamento de Farmacia Hospitalaria, Hospital Universitario Nuestra Señora de la Candelaria, 38010 Santa Cruz de Tenerife, Spain; 4 Unidad de Investigación, Hospital Universitario Nuestra Señora de la Candelaria, Instituto de Investigación Sanitaria de Canarias (IISC), 38010 Santa Cruz de Tenerife, Spain; 5 Laboratorio de Fisiopatología Renal, Departamento de Nefrología, Instituto de Investigación Sanitaria Gregorio Marañón, Hospital General Universitario Gregorio Marañón, 28007 Madrid, Spain; mangeleg@ucm.es (M.Á.G.-N.); elena.vazquez.ogando@hotmail.com (E.V.-O.); alberlaz@ucm.es (A.L.); 6 Departamento de Fisiología, Facultad de Medicina, Universidad Complutense de Madrid, 28040 Madrid, Spain; 7 Departamento de Matemáticas, Estadística e Investigación Operativa, Universidad de La Laguna, 38206 San Cristóbal de La Laguna, Spain; frosag@ull.edu.es; 8 Departamento de Medicina Interna, Dermatología y Psiquiatría, Facultad de Medicina, Universidad de La Laguna, 38200 San Cristóbal de La Laguna, Spain; 9 Servicio de Oncología Médica, Hospital Universitario Hospiten Rambla, Grupo Hospiten, 38001 Santa Cruz de Tenerife, Spain

**Keywords:** dorsal root ganglion, oxaliplatin, neurotoxicity, DRG-inflammation, DPEP1, cilastatin, allodynia

## Abstract

Dorsal root ganglion (DRG) neuroinflammation is a hallmark feature of oxaliplatin-induced peripheral neuropathy (OIPN), a dose-limiting side effect with poorly understood molecular mechanisms. In this study, we investigated the protective potential of cilastatin (CIL), a dipeptidase 1 (DPEP1) inhibitor, while elucidating the regulatory roles of FK506-binding protein 51 (FKBP51) and DPEP1 in OIPN pathogenesis. Behavioral allodynia was evaluated using the acetone test in an oxaliplatin (OxPt) rat model. Lumbar DRGs were imaged via advanced confocal microscopy (Zeiss LSM980 Airyscan 2, Zeiss, Oberkochen, Germany) and analyzed using Fiji 1.54t software to determine fluorescence intensity, subcellular distribution, and co-localization metrics (Pearson’s and Manders’ coefficients). OxPt administration induced marked downregulation and cytoplasmic-to-nuclear translocation of FKBP51 within DRG neuronal and glial compartments. Notably, FKBP51 frequently co-localized with DPEP1 and the pro-inflammatory cytokines TNF-α and IL-6. DPEP1 was strongly expressed in DRG neurons, where it exhibited enhanced perinuclear localization. Concomitant CIL treatment significantly reversed these alterations by attenuating the OxPt-induced cell-type- and compartment-specific changes in FKBP51 and DPEP1 distribution, suppressing TNF-α and IL-6 expression, and preventing GFAP overexpression. These findings identify a novel interplay between FKBP51 and DPEP1 within the DRG during OIPN-elicited neuroinflammation, potentially linking stress protein signaling to pro-inflammatory cytokine activation. Consequently, CIL represents a promising therapeutic candidate to prevent chemotherapy-induced neurotoxicity.

## 1. Introduction

Oxaliplatin (OxPt)-induced peripheral neuropathy (OIPN) is a frequent and dose-limiting complication of OxPt-based chemotherapy and represents a subset of chemotherapy-induced peripheral neuropathy (CIPN), a neuropathic pain syndrome arising from injury to the somatosensory system [1,2]. Unlike motor neuropathies, CIPN predominantly affects sensory pathways, manifesting as paresthesia, dysesthesia, and tactile dysfunction [3,4,5,6], while motor involvement remains minimal [7,8]. OIPN develops in two distinct phases. The acute form emerges shortly after infusion, is transient, and frequently triggered by cold stimuli, producing jaw stiffness, pharyngolaryngeal paresthesia, and cold hypersensitivity. Conversely, the chronic form results from cumulative exposure, is long-lasting, and often needs chemotherapy discontinuation due to persistent neuropathic pain and impaired quality of life [9,10,11]. A defining pathological hallmark of OIPN is the selective accumulation of OxPt within dorsal root ganglia (DRG), facilitated by the absence of a blood-nerve barrier and the presence of fenestrated capillaries. Cellular entry occurs through passive diffusion and metal transporters [12]. Within DRG neurons, OxPt targets nuclear and mitochondrial DNA, forming interstrand, intrastrand, and DNA–protein cross-links. OxPt disrupts mitochondrial homeostasis by altering ion flux, promoting calcium overload, elevating reactive oxygen species (ROS), and compromising ATP generation, ultimately driving apoptosis and axonal degeneration [13,14,15,16]. Despite its clinical relevance, therapeutic strategies to prevent or reverse OIPN remain scarce. A deeper understanding of the inflammatory mechanisms underlying OIPN is therefore critical for the development of effective neuroprotective interventions.

Differential gene expression profiling in OxPt-treated patients revealed FK506-binding protein 51 (FKBP51) as one of the genes with pronounced change in the expression level between individuals with and without OIPN (2.7-fold decrease, *p* < 0.0001) [17]. Furthermore, FOLFOX chemotherapy caused a decrease in FKBP51 gene expression in colorectal cancer (CRC) and in metastatic liver tissue resected after OxPt-based chemotherapy, compared to CRC samples from patients before antineoplastic treatment [18].

FKBP51 is a 51-kDa protein that belongs to the immunophilin family, a group of proteins characterized by a specific and relatively conserved sequence of peptidyl-prolyl-(cis/trans)-isomerase (PPIase) domain [19], which is essential for steroid receptor heterocomplexes [20]. From the 16 human FKBP proteins, FKBP51 is tightly related to corticosteroid effects for glucocorticoid receptor (GR) folding biding role [21], regulation of the hormone biding affinity and nuclear translocation of GR [22]. The functions of FKBP51 extend beyond GR regulation to include immune responses, autophagy, epigenetics, apoptosis, cell growth, cytoskeleton dynamics, and metabolism [23,24,25,26,27,28,29]. In the absence of a ligand, GRs remain inactive in the cytoplasm, while ligand binding promotes the assembly of a chaperone complex containing either FKBP51 or FKBP52 [22]. Activated GRs translocate to the nucleus to regulate various immune, cardiovascular, reproductive, nervous, and metabolic processes [18,30,31,32]. FKBP51 negatively regulates GR activation, providing feedback control of glucocorticoid sensitivity [33].

Previous studies suggest that FKBP51 plays a role in cisplatin toxicity in renal tubules and in the protective effect of cilastatin (CIL) by inhibiting dipeptidase-I (DPEP1) [34,35,36]. In parallel, we recently described the expression and subcellular distribution of DPEP1 in DRG from rats, under both control and post-OxPt conditions [36]. DPEP1 localization in neurons, satellite glial cells, and vascular endothelium highlighted DPEP1 as a potential novel mediator of OIPN pathogenesis [37,38,39,40].

In this study, therefore, we sought to characterize the involvement of FKBP51 and DPEP1 in the DRG during OIPN-induced neuropathy, exploring their interaction and assessing the neuroprotective potential of CIL [34,35,36].

DPEP1 is a zinc-dependent, glycosylated homodimer originally identified in renal proximal tubule brush borders [34,41]. DPEP1 hydrolyzes a variety of dipeptides, including carbapenem antibiotics, and participates in glutathione (GSH) turnover, leukotriene metabolism, and lipid regulation [41,42,43]. In GSH catabolism, DPEP1 cleaves Cys-Gly, influencing antioxidant capacity and susceptibility to ferroptosis [44,45,46]. Pharmacological inhibition by CIL preserves GSH and prevents cisplatin-induced ferroptosis [35,41]. Furthermore, DPEP1 metabolizes leukotriene D4, modulating vascular permeability in several organs [47,48]. Beyond enzymatic functions, DPEP1 acts as an adhesion receptor for neutrophils, promoting inflammatory responses [48]. It has also been implicated in tumorigenesis: overexpression enhances colorectal and hepatoblastoma growth through c-Myc and PI3K/Akt/mTOR signaling [49,50], while in other contexts, such as pancreatic and breast cancer, its expression may suppress invasive capacity [51,52]. Expression patterns vary widely across tissues, including kidney, lung, liver, and testis, and the protein localizes to membranes, cytosol, and nuclei [42,53].

This study provides behavioral and confocal evidence showing that FKBP51 and DPEP1 undergo redistribution across neuronal, glial, and endothelial compartments during OxPt-induced inflammation. Their concurrent expression dynamics with those of TNFα and IL-6 indicate an involvement in neuroinflammatory pathways. Importantly, CIL attenuated these effects, supporting its potential as a neuro-glio-vascular stabilizing agent of DRG microenvironments. Collectively, these findings identify FKBP51 and DPEP1 as modulators of OIPN and establish CIL as a promising candidate for neuroprotection in OxPt chemotherapy.

## 2. Materials and Methods

### 2.1. Subjects

All experimental procedures were conducted in accordance with current regulations for the protection of animals used in research: RD 118/2021 (23 February), Law 32/2007 (7 November), and ECC/566/2015 (20 March). Adult male Wistar rats (~250 g) were obtained from the Animal Facility of Instituto de Investigación Sanitaria Gregorio Marañón, Hospital General Universitario Gregorio Marañón (HGUGM, Madrid, Spain). Animals were housed in pairs in conventional cages with free access to food and water under controlled temperature (22 ± 2 °C) and humidity (45–65%). Male rats were selected based on evidence of sex dimorphism in rodent models of inflammatory pain regulation and immune cell signaling in neuropathic pain [54,55], consistent with previous CIPN models [56,57,58].

### 2.2. Animal Model

Rats were randomly assigned to four groups: (1) OxPt (n = 6), (2) OxPt + CIL (n = 6), (3) control (n = 4), and (4) CIL (n = 4). OxPt was provided by the HGUGM Pharmacy Service (5 mg/mL in 5% glucose solution; Braun Medical S.A., Barcelona, Spain) and administered intraperitoneally at a single dose of 6 mg/kg on day 0. Controls received vehicle injections under identical conditions. CIL (ACS DOBFAR, Tribiano, Milan, Italy), an inhibitor of renal DPEP1, was prepared at 150 mg/kg in 0.5 mL of 0.9% saline and injected intraperitoneally immediately after OxPt administration and subsequently every 24 h. The dosing regimen was based on literature and prior group experience [59]. Animals were weighed daily for dose adjustment and behavioral allodynia monitoring. On day 6, rats were euthanized under sevoflurane anesthesia (5% induction, 2% maintenance; Abbvie, Madrid, Spain). After weighing and surgical preparation, blood was collected by cardiac puncture, and tissues were harvested. Lumbar DRG and sciatic nerves were carefully dissected using microsurgical techniques, placed briefly in paraformaldehyde (PFA), fixed in PFA for 24 h, and transferred to 70% ethanol (VWR, Radnor, PA, USA). While immersion fixation can risk hypoxia-induced artifacts, the small size of the DRG (0.5–1.5 mm) ensures rapid fixative penetration. To confirm true biological localization, we analyzed Na+/K+ ATPase subunit isoforms as internal controls. These membrane proteins showed sharp, compartmentalized immunoreactivity without artifactual leakage, directly replicating patterns previously validated by our group [60].

#### Behavioral Assessment of Allodynia

Cold allodynia was assessed using the acetone test [59,61]. A 200 μL droplet of acetone (PanReac, Barcelona, Spain) was applied to the plantar surface of one hind paw using an insulin-type syringe (B. Braun Medical S.A., Madrid, Spain). Left and right paws were alternated to minimize habituation. Behavioral responses (paw withdrawals, shakes, or licks) were recorded over 2 min. Each paw was evaluated three times at 1 h intervals, and the median value of the three measurements was used for analysis, Figure 1.

### 2.3. Antibodies

The antibodies used in this study are listed in Table 1. Localizations of providers were as follows: Abcam, Cambridge, UK; Santa Cruz Biotechnology, Dallas, TX, USA; and Sigma-Aldrich, St. Louis, MO, USA. All antibody incubations were performed according to the manufacturers’ instructions. Table 1 lists the antibodies used, their species of origin, dilution, source, and catalog number, as well as the figures in this article showing the results obtained with them.

### 2.4. Immunohistochemistry and Immunofluorescence 

DRG tissues were embedded in paraffin and sectioned (5 μm). Sections were deparaffinized, rehydrated, and subjected to antigen retrieval in sodium citrate buffer (pH 6.0) at 120 °C for 10 min. Non-specific binding was blocked with 5% BSA or serum in Tris-buffered saline (TBS) for 1 h at room temperature. Sections were incubated overnight at 4 °C with primary antibodies, and negative controls (omission of primary antibodies) were included. Secondary antibodies were applied for 1 h at room temperature in the dark. Slides were mounted with ProLong^®^ Diamond Antifade Mountant with DAPI (Invitrogen, Carlsbad, CA, USA; Cat. P36965).

### 2.5. Confocal Microscopy

Images were acquired using a Zeiss LSM980 Airyscan-2 (Zeiss, Oberkochen, Germany). Raw files (CZI or LIF) were exported as JPEGs (300 dpi). Figures were assembled in Adobe Photoshop CC 2018.

### 2.6. Quantitative Image Analysis

Confocal images were analyzed using the Fiji software (ImageJ version 1.50i). To quantify the signal of DPEP1 and FKBP51 molecules, 40× magnification images were used, and fluorescence intensity was measured in each cell, both in the cytoplasm and in the nucleus. Fluorescence intensity quantification was made possible by the design of Regions of Interest (ROIs). In each image, regions of interest (ROIs) were classified as follows: first, ROIs were created for each cell nucleus, and second, ROIs were created to fit the cytoplasm of each cell. Due to the variability in cell size in each image, it was decided to design different sets of ROIs with different cell sizes for each image (6 large ROIs, 6 medium ROIs, and 18 small ROIs). Additionally, single-cell quantification of DPEP1 and FKBP51 was performed specifically in satellite glial cells. The global fluorescence intensity of GFAP and TNFα was also quantified to assess the degree of glial cell reactivity and the inflammatory state of the tissue. Due to the type of staining, this quantification was performed by creating a large region of interest (ROI) within the image and a small region located in a dark area to quantify the background and subtract it from the fluorescence intensity measurement of interest. A fluorescence intensity threshold was applied to minimize background noise, such that anything below the chosen threshold was considered “0”. This approach allowed for a more precise and accurate quantification of the actual fluorescence intensity of the molecules of interest. Each region of interest (ROI) has a number, and its location in the image is always visible, making it impossible to measure an ROI more than once. All images used were taken at 40× magnification, and between 10 and 35 measurements were taken per photograph. For the DPEP1- FKBP51 colocalization analysis, photographs in the green channel for DPEP1 and the red channel for FKBP51 were used, and the Fiji Coloc Threshold plugin was used. The software applied an automatic thresholding method known as cost thresholding, which distinguishes between background noise and actual colocalization. Signals above the threshold were considered actual colocalization, while signals below the threshold were considered background noise. From this analysis, several parameters were calculated, including Pearson’s and Manders’ coefficients (PCC and MCC, respectively), as well as the actual colocalization percentages between the two channels [62,63]. Further information on the procedure and analysis is provided in the Supplementary Materials.

### 2.7. Qualitative and Semi-Quantitative Image Analysis

Being aware of the possible errors in the use of plugins [62], in addition to quantitative analysis, blinded qualitative and semi-quantitative evaluations were performed, although in this report we will only present the results of the quantitative analysis. Two independent observers graded staining intensity as absent (0), basal or faint (½), low (1), moderate (2), or strong (3). Cutoffs were established by consensus after pilot review. Discrepancies of >1 unit were averaged. Results are provided in Table S1 in Supplementary Material S1.

### 2.8. Statistical Analysis

Statistical analyses were performed using SPSS v29.0 (IBM, Armonk, New York, NY, USA). Data are expressed as mean ± standard error of the mean (SEM). Normality was tested with the Kolmogorov–Smirnov test, and variance homogeneity with Levene’s test. Normally distributed data were analyzed by analysis of variance (ANOVA) with Newman–Keuls post hoc tests or by Student’s *t*-test for two-group comparisons. Non-parametric data were analyzed using Kruskal–Wallis tests. Behavioral data from the acetone test were analyzed by one-way ANOVA. A *p*-value < 0.05 was considered statistically significant. Complementary information about the quantitative statistical analysis of this study is given in Supplementary Material S2. 

## 3. Results

### 3.1. CIL Prevented Allodynia Elicited by OxPt

To evaluate the effect of CIL on OxPt-induced mechanical allodynia, the mean number of pain responses (hind paw withdrawals, shakings, or lickings) was determined both on a daily basis over the 6-day study period (Table 2) and globally throughout the entire model (Figure 1). One-way ANOVA revealed that control groups (Control and CIL) showed no allodynic responses at any time point during the study (0.00 ± 0.00). In contrast, OxPt administration (OxPt) induced a progressive increase in mechanical pain starting from day 1 (1.67 ± 0.84), reaching statistically significant differences compared to control groups from day 2 onwards (4.33 ± 2.19, *p* ≤ 0.0578) and peak levels on days 3 (6.67 ± 1.12) and 5 (12.17 ± 2.53, *p* ≤ 0.0169 vs. all groups). Co-administration of CIL (OxPt + CIL) substantially prevented this effect, maintaining markedly reduced allodynia scores throughout the daily follow-up (*p* ≤ 0.0053 on day 3 vs. Control ± CIL, and *p* ≤ 0.0169 on day 5 vs. OxPt). Consistent with these daily findings, analysis of the overall cumulative mean for the model confirmed that the group treated with OxPt alone exhibited a significant increase in overall mean allodynia compared to all other groups (*p* ≤ 0.0052 vs. all groups), whereas co-administration with CIL effectively prevented this cumulative increase (Table 2 and Figure 1B).

### 3.2. FKBP51 and DPEP1 Cellular and Subcellular Distribution in DRGs from Control, OxPt, and CIL Treated Rats

In control samples, the FKBP51 immunostaining signal was detected at a medium level in a remarkable dot-like cytosolic pattern of neurons and glial cells (white stars). DPEP1-specific immunofluorescence was observed at higher intensity in the neuronal cytosol in a reticulated fashion, while its expression in satellite glial cells reached only medium levels, creating a distinct interphase between both cell types (yellow arrowheads, first horizontal lane in Figure 2). Confocal images showed a moderate-to-high spatial colocalization between DPEP1 and FKBP51 in different ganglionic regions (PCC = 0.413 ± 0.050; Supplementary Material S1, Figure S1), including neurons, DRG capsule (orange arrows), and endothelial and satellite glial cells, where the brightest FKBP51 signal was consistently evident (magenta arrowhead).

In DRGs from CIL-treated rats, changes in FKBP51 subcellular distribution were noticed, with increased localization of this immunophilin inside neuronal nuclei, while endothelial and satellite glial cells displayed brighter cytosolic signals in dot-like fashion (white arrowheads), particularly near the nuclear envelope. A heterogeneous reticulated pattern of DPEP1 immunofluorescence, like controls, was also observed, with a medium-to-high intensity signal in axons of the root (yellow star; Figure 2). Co-expression of FKBP51 and DPEP1 was detectable in neurons but was substantially lower than in controls, with a lower mean PCC and considerable cell-to-cell variability (PCC = 0.175 ± 0.120; Supplementary Material S1, Figure S1). Signal was predominant for DPEP1 in the cytosol and for FKBP51 in the nuclei. Notably, spherical nuclear structures devoid of FKBP51 but with low DPEP1 intensity were detected (magenta arrows).

Under OxPt stress conditions, neurons showed a high degree of damage and vacuolization in a homogeneous manner, regardless of the size or type of neuron. DRGs showed remarkable FKBP51 expression and distribution, at lower intensity than in controls or in CIL-treated rats. A moderate-intensity of FKBP51 signal was found predominantly in neurons and satellite glial cells, with certain neurons displaying slightly brighter dot-like cytosolic signals (white arrowheads). However, overall, FKBP51-specific immunofluorescence appeared reduced in cytosol and nuclei across cell types, with only specific cytosolic regions retaining higher density. DPEP1 immunostaining was detected at medium-to-high levels in neurons and glial cells, showing a reticulated cytosolic pattern and medium nuclear intensity (magenta arrows). Despite the overall reduction in FKBP51 signal, the spatial association between FKBP51 and DPEP1 was intermediate between that observed in control and CIL-treated samples, with a mean PCC of 0.327 ± 0.146 (Supplementary Material S1, Figure S1). Thus, OxPt treatment did not increase colocalization relative to controls; rather, the PCC was lower than in controls but higher than in CIL-treated samples, with the relatively large standard deviation indicating substantial variability among samples/cells. On the other hand, a reticulated pattern of FKBP51 was found in some neurons cytosol, while in others there was a homogenous dot pattern.

In OxPt + CIL-treated rats, FKBP51 and DPEP1 distributions resembled control conditions. Medium-to-low FKBP51 immunofluorescence was observed in the root (magenta star), while endothelial and satellite glial cells displayed higher FKBP51 intensity (yellow arrowheads), localized in cytosolic dot-like fashion and also within neuronal nuclei (white arrow), similar to CIL-treated group. DPEP1 expression appeared at medium-to-high levels, heterogeneously distributed in neurons, glial cells, and axonal roots, maintaining its basal reticulated pattern as in controls (Figure 2). The spatial co-localization of FKBP51 and DPEP1 was highest among the four experimental conditions (PCC = 0.452 ± 0.053; Supplementary Material S1, Figure S1), being predominant in the cytosol of neurons and satellite glial cells.

### 3.3. FKBP51 and DPEP1 Expression in Satellite Glial Cells of DRGs from Control, OxPt, and CIL Treated Rats

With the aim of studying FKBP51 expression in glial cells, co-immunostaining images with GFAP were obtained, Figure 3. In control panels, the FKBP51 immunostaining signal revealed the presence of this immunophilin in glial cells. Presumably, augmented co-expression of FKBP51 and GFAP was found in CIL-treated rat DRG samples, where the immunostaining signal of FKBP51 showed a similar pattern and intensity to the control panels, and GFAP immunostaining signal was present colocalizing with FKBP51 in some glial cells cytosol (PCC = 0.387 ± 0.063). In this row of images, there were presumably Schwann cells accompanied by axons at the root and showed FKBP51 expression in the same way as glial cells at the ganglion body. Notably, low or weak FKBP51 expression was observed in the nuclei of neurons and glial cells of the DRGs of subjects treated with OxPt. However, the most intense FKBP51 immunostaining signal was observed in some glial cell nuclei. A high-intensity GFAP signal was evident in glial cells surrounding the axons from the root. Satellite glial cells around neurons depict the GFAP signal along with light FKBP51-specific staining.

Samples of OxPt + CIL-treated rats presented an evident immunofluorescence signal for FKBP51 in most neurons’ nuclei with an absent signal in spherical spaces inside some nuclei. The FKBP51 signal was also brighter inside nuclei than cytosol in glial cells, pointed by magenta arrowheads in Figure 3, similar in intensity to the control and CIL-treated group. GFAP immunofluorescence was at variable intensity in satellite glial cells colocalizing with the FKBP51 signal in cytosol.

Total GFAP intensity per image in 40× pictures, measured by ImageJ-FIJI (Supplementary Material S1, Figure S2) in a total of 10 images per group, resulted in control: 37.8 × 10^6^ arbitrary units (au), CIL: 22.8 × 10^6^ au, OxPt: 260.8 × 10^6^ au and OxPt + CIL 20.2 × 10^6^ au. These quantifications confirm the overexpression of GFAP following OxPt administration that is avoided by the coadministration of CIL with the chemotherapeutic.

Quantitative analysis of FKBP51 and DPEP1cell by cell of satellite glia in 40× images, measured by ImageJ-FIJI (Supplementary Material S1, Figure S3) in a total of 30 cells in 10 images per group, resulted in the following intensity values: for FKBP51, control: 119.2 ± 52.2 × 10^3^ au, CIL: 140.1 ± 69.1 × 10^3^ au, OxPt: 70.4 ± 36.1 × 10^3^ au and OxPt + CIL 120.1 ± 49.0 × 10^3^ au. DPEP1 intensity values: control: 268.1 ± 53.3 × 10^3^ au, CIL: 208.5 ± 78.2 × 10^3^ au, OxPt: 171.4 ± 59.1 × 10^3^ au and OxPt + CIL 263.5 ± 50.5 × 10^3^ au. Specifically, quantitative analysis of satellite glial cells showed significantly lower FKBP51 and DPEP1 immunofluorescence intensities in the OxPt-treated group (*p* < 0.05), whereas no significant differences were observed among the Control, CIL, and OxPt + CIL groups.

### 3.4. FKBP51 Expression in Endothelium of DRG

The expression and distribution of FKBP51 in the structure of DRG blood vessels was studied using the CD31 marker. In the control panels of Figure 4, CD31-specific immunofluorescence signal was present at moderate intensity around neurons and close to satellite glial cells in the ganglion body and more abundant at higher levels in endothelial cells surrounding the axons and accompanying the Schwann cells and endoneurium and perineurium along the root. 

In CIL-treated animals, DRG showed areas near neurons and glial cell bodies showing CD31 signal in co-localization with FKBP51 more remarkably than in the root, mainly in the cytosol of endothelial cells (PCC = 0.278 ± 0.111).

Effects of OxPt on CD31 expression were slightly noticeable, with an increase in specific immunostaining in glial cells from the root of axons. A low-intensity, barely visible signal was found for the CD31 signal around neurons and glial cells from the ganglion. The specific FKBP51 signal appeared in a heterogeneous reticulated pattern in neurons and glial cells cytosol in a medium level with brighter intensity in nuclei, as well as in glia cells from the root of axons (OxPt lane in Figure 4). In OxPt + CIL-treated animals, several endothelial cells in the DRG showed CD31 immunostaining in a network fashion at the root of axons next to the ganglion and a low signal of CD31 adjacent to neurons’ bodies and satellite glial cells.

### 3.5. Effects on FKBP51 Expression in the Short Time DRG Inflammation Response to OxPt Chemotherapy

To analyze the involvement of FKBP51 in the inflammatory response of DRG after a single dose of OxPt, pro-inflammatory molecules TNFα and IL-6 were used as markers, Figure 5 and Figure 6. In control samples, TNFα immunostaining signal (Figure 5) was low or virtually absent in neurons and at low intensity (basal) in some glial and endothelial cells. IL-6 expression was found at a low level in neurons and at low intensity in some glial cells in the ganglion body and axons from the root (Figure 6).

CIL-treated rat samples showed higher fluorescence intensity for TNFα and IL-6 and the same localization pattern as in controls. A low level of TNFα immunofluorescence signal was shown in the cytosol of neurons and axons forming the root, while a higher intensity signal was present in glial and endothelial cells. IL-6 varied from medium to high intensity levels in endothelial and glial cells and low or faint in neurons’ cytosol. In both Figure 5 and Figure 6, fluorescence for FKBP51 was at a brighter intensity than control panels and at a higher intensity level in the nuclei of some neurons in the fashion of spark-points FKBP51-signal.

Images for FKBP51-specific staining in DRGs from OxPt-treated rats showed homogeneous lower fluorescence intensity within the damaged neurons, glia, and cells in the root. TNFα-specific fluorescence depicted a heterogeneous cellular pattern varying from very high to low intensity levels and variable PCC among neurons (PCC = 0.358 ± 0.308), merged in Figure 5, OxPt line. Satellite glial cells exhibit TNFα fluorescence levels in the high range of intensity and scarce spots or nothing in tracts of the root. 

In DRG samples from rats treated with OxPt + CIL, the specific fluorescence pattern either for FKBP51, TNFα, or IL-6 were similar to those from the group treated with CIL alone.

To evaluate the average of the inflammation state, total TNFα intensity per image in 40× pictures was measured by ImageJ-FIJI (Supplementary Material S1, Figure S4) in a total of 10 images per group, resulted in control: 11.3 × 10^6^ au, CIL: 35.8 × 10^6^ au, OxPt: 67.8 × 10^6^ au and OxPt + CIL 32.2 × 10^6^ au. The variations in these values confirm the effect of CIL in preventing inflammation caused by OxPt.

### 3.6. Quantitative Analysis and Modeling of FKBP51 and DPEP1 Expression

#### 3.6.1. Statistical Refinement of Results

Allodynia Prevention: One-way ANOVA revealed that while the OxPt-only group exhibited a pronounced increase in hind paw withdrawal, shaking, and licking (*p* < 0.0052 vs. all groups), co-administration with CIL (OxPt + CIL) significantly attenuated this effect, keeping overall allodynia levels markedly lower throughout the study model (Figure 1).

Protein Interaction: Protein colocalization analysis showed treatment-dependent differences in the spatial relationship between FKBP51 and DPEP1. Although the PCC was apparently lower in the OxPt group (0.327 ± 0.146) compared with the control group (0.413 ± 0.050), the number of colocalization events per field (intensity of colocalized fluorescence) increased following OxPt treatment and was subsequently stabilized in the OxPt + CIL group (PCC = 0.452 ± 0.053). See Table S1 and Figure S5 in Supplementary Material S1.

Glial Cells Reactivity Quantification: GFAP overexpression in the OxPt group was quantified at 260.8 × 106 au, an almost 7-fold increase compared to controls, which was almost completely suppressed by CIL (20.2 × 10^6^ au).

Figure 7 shows a summary that compiles the results of the proteins and markers studied in the form of histograms.

#### 3.6.2. Modeling FKBP51 and DPEP1 Expression and Variability in Rat DRG Neurons

For this study, fluorescence measurements were obtained from individual cells, and the Mean Fluorescence Intensity (MFI) was used as the primary dependent variable. To account for morphological differences among the cells, they were categorized into three size groups: Small, Medium, and Large.

The current analytical approach directly models the MFI while incorporating cell size as a fixed categorical factor, which effectively controls for size-related variations while preserving the original measurement scale. Furthermore, because multiple cells are measured within the same field of view, the data have a nested structure. To account for this and to control for potential technical baseline shifts or illumination variability between acquisitions, the specific image from which the cells were extracted was included as a random effect. This approach provides a robust evaluation of the experimental conditions while properly handling the hierarchical nature of the imaging data.

Importantly, because exactly one image was acquired and analyzed per individual rat in this in vivo study, the ‘Image’ random effect directly corresponds to the biological experimental unit (the rat). This appropriately accounts for inter-animal biological variability and prevents pseudo replication, as there is no clustering of multiple images within the same animal.

Therefore, the linear mixed model applied for each protein (FKBP51 and DPEP1) independently can be expressed by the following equation:

$$Y_{ijkl}=\mu +\;\alpha_{i}+\;\beta_{j}+{\left(\alpha \beta \right)}_{ij}+\tau_{k}+\varepsilon_{ijkl}$$

In which:

- ***Y_ijkl_*** represents the *MFI* of the *l*-th cell, measured in the *k*-th image, under the *i*-th treatment and *j*-th cell size category
- ***μ*** is the overall intercept (grand mean)
- ***α_i_*** is the fixed effect of the *i*-th Treatment (Control, CIL, OxPt, OxPt + CIL)
- ***β_j_*** is the fixed effect of the *j*-th Cell Size (small, medium, large)
- (***αβ***)***_ij_*** is the fixed interaction between Treatment and Cell Size
- ***τ_k_*** is the random effect of the *k*-th Image, accounting for the nested structure of the data and technical baseline shifts $\left(\tau_{k}\;\sim \;N\left(0,\sigma_{image}\right)\right)$
- ***ε_ijkl_*** is the residual error term for each individual cell $\left(\varepsilon_{ijkl}\;\sim \;N\left(0,\sigma \right)\right)$

FKBP51 expression analysis: The model revealed no significant main effects of Treatment (*F* = 2.595, *p* = 0.101) or Cell Size (*F* = 0.035, *p* = 0.966) on FKBP51 fluorescence intensity. However, the Treatment x Cell Size interaction was statistically significant (*F* = 2.900, *p* = 0.009), indicating that the effect of treatment on FKBP51 expression is modulated by cell size. Post-hoc analyses for this interaction revealed that the CIL treatment significantly altered FKBP51 fluorescence intensity specifically in small cells (*p* = 0.031), whereas no significant treatment effects were observed on medium or large cells.

DPEP1 expression analysis: A linear mixed model was performed to evaluate the effect of the treatments on DPEP1 expression, using the Mean Fluorescence Intensity as the dependent variable, with Treatment and Cell Size as fixed factor, and Image as a random effect. The analysis revealed significant main effects for both Treatment (*F*(3, 11.16) = 8.250, *p* = 0.004) and Cell Size (*F*(2, 418.17) = 6.370, *p* = 0.002), indicating that the effect of the treatment on DPEP1 fluorescence intensity is modulated by the cellular size. Post-hoc pairwise comparisons against the control group revealed that both CIL and OxPt treatments significantly decreased DPEP1 fluorescence intensity across all cell sizes (small, medium, large; all *p* < 0.05). In contrast, the combined OxPt + CIL treatment did not significantly alter DPEP1 levels compared to the control in any cell size category (all *p* > 0.05). 

Tables S1–S5 in Supplementary Material S2 summarize and complement these results.

## 4. Discussion

Our study provides novel insights into the pleiotropic cellular and subcellular-specific responses of FKBP51 and DPEP1 in DRG under OxPt-induced neurotoxicity and its modulation by CIL. By integrating cold-evoked nocifensive behavior with the cellular and subcellular distribution of FKBP51, DPEP1, TNFα, and IL-6 across neurons, GFAP-positive glial cells, and CD31-positive endothelial cells, we observed complex patterns of response and potential compensatory mechanisms. Furthermore, CIL did not just inhibit DPEP1 activity; it appeared to stabilize the DRG microenvironment, at least during the short term of our experimental model. In rats treated with OxPt + CIL: FKBP51 levels remained closer to baseline (control) levels, the massive increase in the marker GFAP was avoided, and proinflammatory cytokine expression was significantly reduced [64].

### 4.1. Disruption of DPEP1 and FKBP51 Protein Dynamics as a Mechanism of OxPt Neurotoxicity

Our immunofluorescence analysis reveals that OxPt-induced neurotoxicity manifests as a disruption of protein dynamics within the DRG neurons, satellite glia, and endothelial cells. The transition from a basal “reticulated” DPEP1 and “dot-like” FKBP51 patterns to alter signal intensity and subcellular redistribution under OxPt stress suggests disruption of intracellular protein trafficking and compartmentalization. CIL co-treatment partially restored these distribution patterns, indicating a protective effect on FKBP51 and DPEP1 subcellular organization [19,65]. These changes in protein dynamics likely impair the neurotrophic coupling and signal turnover between glial cells and neurons. CIL appears to preserve these distribution patterns, maintaining the balanced subcellular immunofluorescence distribution of FKBP51 between the nuclear and cytosolic compartments and the structural organization of DPEP1, while simultaneously preventing an increase in inflammatory markers (TNFα, IL-6) [66,67,68].

### 4.2. Involvement of FKBP51 and DPEP1 in OxPt Toxicity and Neuroinflammation of DRG

In control DRGs, FKBP51 displayed a characteristic cytosolic dot-like pattern in neurons (Figure 2), consistent with its known roles in protein folding and GR regulation. Lower nuclear FKBP51 expression in glia and neurons reflects a basal state of cellular homeostasis. The spatial overlap of DPEP1 with FKBP51 in neurons, glia, and endothelial cells suggests involvement in metabolic regulation and vascular function, even under physiological conditions. The low TNFα and IL-6 signals are consistent with the absence of overt inflammatory activation under basal conditions.

CIL alone largely preserved baseline FKBP51 and DPEP1 distribution, though it induced compartment-specific enhancements in neurons and glia compared to controls, although a modest increase in nuclear FKBP51 was observed, accompanied by slightly enhanced colocalization with DPEP1 in neuronal cytoplasm and glial nuclei (Figure 3). The lack of increase in inflammatory markers (TNFα, IL-6) suggests that CIL does not trigger proinflammatory pathways in the DRG under non-stressed conditions. However, the increase in FKBP51-specific fluorescence signals in endothelial and satellite glial cells (Figure 3 and Figure 4) could indicate an early priming effect or cytoprotective activation in these compartments [69].

Upon OxPt treatment, DRG displayed profound neuroinflammatory and structural alterations. A marked shift was observed in FKBP51 expression, with a reduction of cytosolic levels and prominent nuclear accumulation in glial cells, while neurons displayed a heterogeneous pattern, with some neurons showing FKBP51 nuclear exclusion (Figure 2). This dual pattern could reflect a context-dependent response where FKBP51 acts as a molecular switch between pro- and anti-inflammatory roles. Importantly, this redistribution paralleled the strong induction of TNFα and IL-6 in neurons, glia, and endothelial cells, highlighting a potent neuroinflammatory environment (Figure 5 and Figure 6). Yu et al. have shown upregulation of FKBP51 expression through qRT-PCR in the DRG of rats after chronic constriction injury and demonstrated a link between FKBP51 and the development and maintenance of neuropathic pain [70].

The increased spatial colocalization of FKBP51 and TNFα in neurons suggests that FKBP51 may contribute to or attempt to buffer the proinflammatory milieu induced by OxPt, particularly through NF-κB pathway modulation [71,72] (Figure 5). As mentioned, the FKBP51 characteristic role in steroid hormone receptors is not the only function this immunophilin plays in the metabolism of cells. FKBP51 is a mediator of a huge array of signaling pathways, joined to phosphatases and kinases that are critical in cellular survival and response to external factors. More specifically, the transcription factor NF-κB and its pathway have been related to neuropathic pain and neurological inflammation [73]. This nuclear factor regulates transcription of genes from chemokines, cytokines, and proinflammatory enzymes, among others, in neurons but also in glial and endothelial cells, contributing to the inflammatory environment [73,74]. Romano et al. suggested that FKBP51 contributes to NF-κB activation and resistance of melanoma cells to apoptosis through the IKK complex and its catalytic ability [72]. Similar results were obtained by Jiang et al. in glioma cell survival [71]. Nevertheless, in these studies FKBP51 expression was analyzed via RT-PCR and Western-blot without specific information on the cellular and subcellular localization of this protein. Our results provide innovative information in these pathways by demonstrating distinct, cell-type-specific changes in FKBP51 dynamics. Under stress conditions, glial cells exhibited increased expression of this immunophilin. In neurons, however, OxPt induced a heterogeneous redistribution of FKBP51—characterized in a subset of cells by a reduced or absent nuclear signal—whereas CIL co-treatment enhanced nuclear FKBP51 localization. Controversial to previous studies [73], the increased level of TNFα was not accompanied by a rise of FKBP51 in neurons. The reason why TNFα rise was not in line with FKBP51 increased expression could be the activation of specific NF-κB dimers, such as the RelA subunit, which depends on the external factor that triggered this pathway’s activation and contributes to cell death after injury and not to cell survival [73,75,76,77].

### 4.3. Differential Expression of FKBP51 and DPEP1 in DRG Cells

The variability of responses within the cellular heterogeneity of the DRG is evident in the merge images of FKBP51 and DPEP1 in samples from rats treated with CIL, OxPt and OxPt + CIL. Heterogeneity in the cellular response is also observed in the different levels of TNFα expression in the neurons of the samples that were treated with OxPt, Figure 5, merge FKBP51 and TNFα.

Endothelial FKBP51 and CD31 expression—maintained at baseline in controls and modestly enhanced without inflammation by CIL alone—experimented OxPt-induced depletion that was markedly restored upon CIL co-treatment. Similarly, satellite glial cells displayed elevated GFAP expression, reinforcing the idea of glial activation as an essential contributor to DRG inflammation and pain sensitization (Figure 3). As well as in neurons, NF-κB pathway is implicated in glia and blood vessel responses to different stimuli and contributes to changes in cellular permeability, endocytosis, and molecular trafficking [73]. Stone et al. showed that brain microvessel endothelial cells responded to TNFα presence, but also to other cytokines such as IL-15, and the final effect in these cells was determined by the proportion effect of each one in NF-κB transcription activity [78].

On the other hand, glia activation triggered by OxPt could be related to neuronal damage, namely satellite glial cell reactivity, but also to its own response to this xenobiotic. In DRGs from OxPt-treated rats (Figure 5), the more pronounced increase in TNF-α in neurons relative to glia, coupled with the altered intracellular distribution and increased nuclear localization of FKBP51, may point to short-term damage and an early stage of satellite glial cell reactivity. 

Remarkably, CIL co-treatment mitigated many of these changes (Figure 3 and Figure 5). FKBP51 expression increased in neuronal and glial nuclei, showing brighter intensity than in OxPt-only animals, while its cytosolic levels were restored partially in neurons. This nuclear accumulation, along with decreased TNFα and IL-6 levels in neurons and glial cells, suggests that CIL fosters a neuroprotective and anti-inflammatory state, potentially through FKBP51-mediated modulation of stress response pathways such as Akt/mTOR, NF-κB, or GR signaling [79,80,81]. Moreover, CIL preserved CD31 expression in endothelial cells and prevented their loss in perineuronal regions as a possible sign of protective vascular effects that might contribute to decreased inflammatory infiltration and edema.

From a cell-type perspective, our data indicate that neuronal FKBP51 may act as a context-dependent modulator of resilience to stress and inflammation, while glial FKBP51 overexpression may reflect both glia reactivity and an attempt to restrain inflammatory responses [64,82].

Unlike the surgical trauma model of Wedel et al. [83]—where delayed SAFit2 treatment inhibits FKBP51 to alleviate established pain via lipid metabolism (C16 dihydroceramide) and TRPV1/CGRP signaling—our chemical model deploys an immediate, preventative strategy using the DPEP1 inhibitor CIL against systemic oxaliplatin toxicity. Rather than blocking active FKBP51 to treat thermal or mechanical defects, CIL rescues chemotherapy-induced FKBP51 effects, thereby suppressing neuroinflammation (TNFα, IL-6), providing a specialized strategy to prevent dose-limiting oncological neurotoxicity.

Together, our results support that FKBP51 acts as a key integrative node in OxPt-induced DRG inflammation through cell-type- and compartment-specific mechanisms, while CIL, a DPEP1 inhibitor, promotes nuclear FKBP51 accumulation to reduce proinflammatory cytokine production. However, while our findings demonstrate that CIL effectively attenuates short-term OxPt-induced neuroinflammation and preserves endothelial CD31 immunoreactivity integrity, these findings only reflect the short-term nature of our experimental design. Extended studies in longer-time neuropathy models are warranted to determine whether CIL offers sustained neuroprotective and microvascular benefits without compromising chemotherapy efficacy.

### 4.4. FKBP51 and DPEP1 Interactions Within Nuclear Protein Networks: STRING-Based Functional Enrichment: Moonlighting Roles and Perspectives on Mechanistic Bases

FKBP51 and DPEP1 are cytosolic proteins that perform their recognized functions within the cytoplasm. Under certain circumstances, however, these proteins undergo nuclear translocation and interact with nuclear proteins. In addition to the NF-κB pathway discussed above, Figure 8, panel A, represents FKBP51 interacting with CDK11A, CDK15, CDK4, CDK9, CDKL1, CLK3, MOS, PIPOX, PSKH2, STK11, ULK3, WDR90 and DPEP1 interacting with SMC2, SMC4, NCAPH, NCAPG, NCAPD2, FANCG, ORC5, SUN1.

The proteins that interact with FKBP51 form a cluster called “Cyclin-dependent protein serine/threonine kinase activity” and most of them are involved in cell cycle regulation, differentiation, and proliferation [84,85]. Briefly, SMC2 (structural maintenance of chromosomes 2), SMC4 (structural maintenance of chromosomes 4), NCAPH (non-SMC condensin I complex subunit H), and NCAPG (non-SMC condensin I complex subunit G) are a regulatory subunit that associates with NCAPG, NCAPD2 (non-SMC condensin I complex subunit D2), and both the N- and C-terminal regions of SMC-4 and SMC-2 proteins, facilitating the formation of the highly conserved SMC condensin I complex in eukaryotes [86,87,88,89]. SMC2, SMC4, NCAPH, NCAPG, and NCAPD2 form a cluster called “+ Positive regulation of chromosome condensation”. FANCG (FA complementation group G) and ORC5 (origin recognition complex subunit 5) [90,91]. SUN1 (Sad1 and UNC84 domain containing 1) SUN1A and SUN2 proteins are critical components of the linker of nucleoskeleton and cytoskeleton (LINC) complex [92,93,94]. Experimental data support that all proteins mentioned previously have interaction probabilities exceeding 0.99. A weaker interaction was observed with TNPO2 (*p* = 0.753) (Transportin 2, a protein transporter between the cell nucleus and cytoplasm) (Figure 8) [84,85]. 

**Figure 8:**
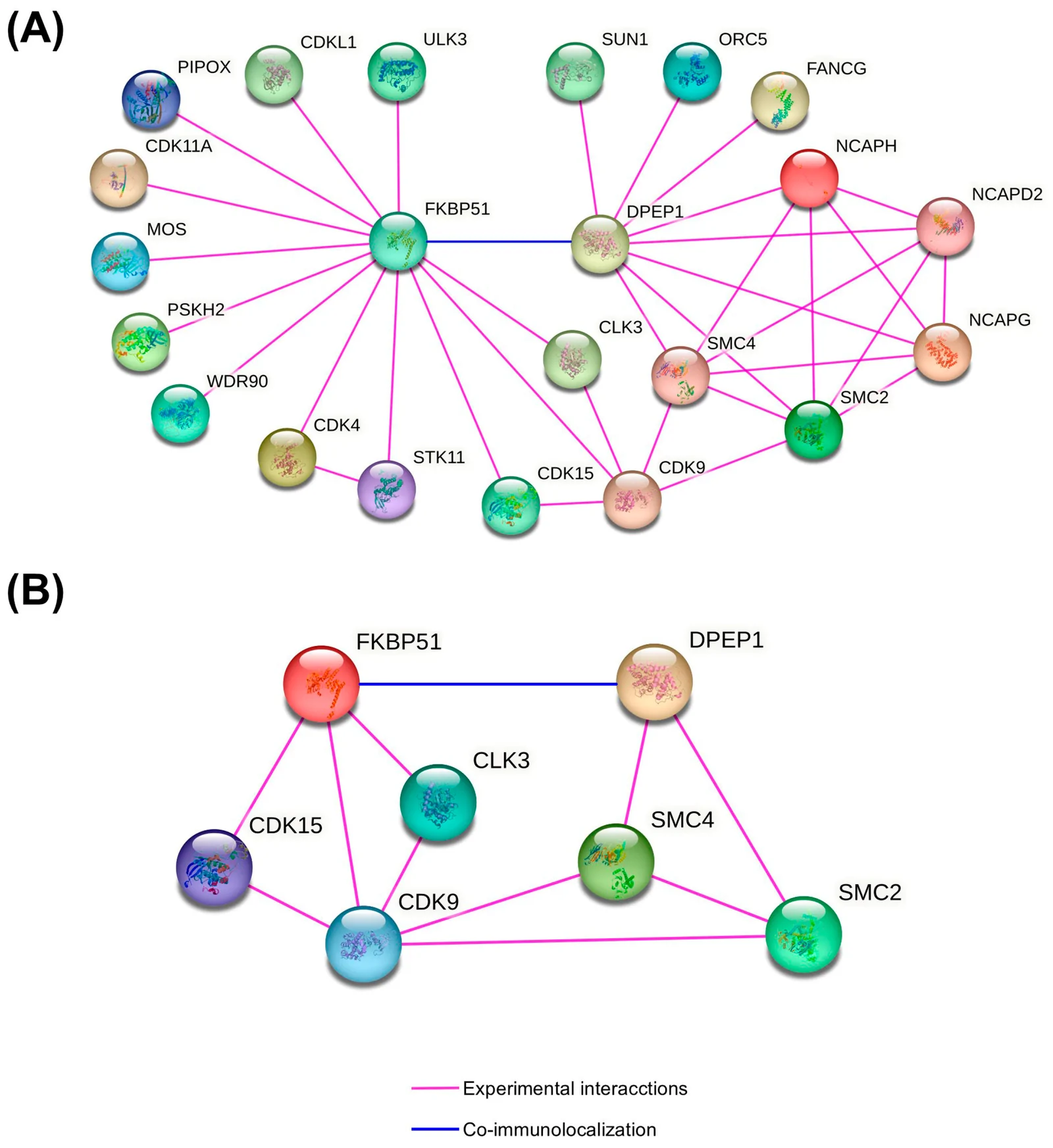
Panel (**A**). FKBP51 and DPEP1-nuclear proteins association networks and functional enrichment analysis using STRING database (https://string-db.org) [95]. Panel (**B**). STRING of experimental interactions of the FKBP51-CDK9-SMC4-DPEP1 protein cluster.

The proteomic analyses reveal protein-protein interactions between FKBP51, DPEP1, and several nuclear proteins that suggest that DNA damage induced by OxPt somehow triggers the translocation of FKBP51 and DPEP1 to the nucleus. Consequently, we hypothesize that nuclear-translocated FKBP51 and DPEP1 function cooperatively as modifiers within the aforementioned interactome to regulate genomic stability. Experimental validation of this hypothesis will establish a framework for future mechanistic studies aimed at elucidating the pathophysiology of OxPt-induced neurotoxicity and its prevention by CIL.

## 5. Conclusions

This study reports the cellular and subcellular localization of FKBP51 and DPEP1 in the DRGs of control rats and those subjected to short-term OxPt treatment (acute injury phase), with or without CIL, using specific well-characterized antibodies. In the DRG, OxPt drives a neuroinflammatory response characterized by cell-type-specific changes in FKBP51 and DPEP1 distribution. Specifically, OxPt alters the nuclear-to-cytoplasmic ratio of FKBP51, inducing increased nuclear accumulation in satellite glial cells without significantly altering total protein abundance, while neurons exhibit a heterogeneous redistribution pattern. Concurrently, DPEP1 undergoes structural and spatial alterations. CIL co-treatment partially restores these distribution patterns, accompanied by reduced TNF-α and IL-6 levels and the preservation of CD31, supporting a protective role across neuronal, glial, and vascular compartments. These results highlight FKBP51 as a potential intracellular mediator of OxPt-induced neuroinflammation and support CIL as a promising therapeutic adjuvant for sensory neuron protection. Collectively, our findings unveil novel molecular targets for mitigating acute OxPt-evoked hypersensitivity; nevertheless, future studies are required to fully elucidate their biological significance, evaluate translational potential, and address possible differences due to sexual dimorphism in inflammatory pain regulation.

## Figures and Tables

**Figure 1:**
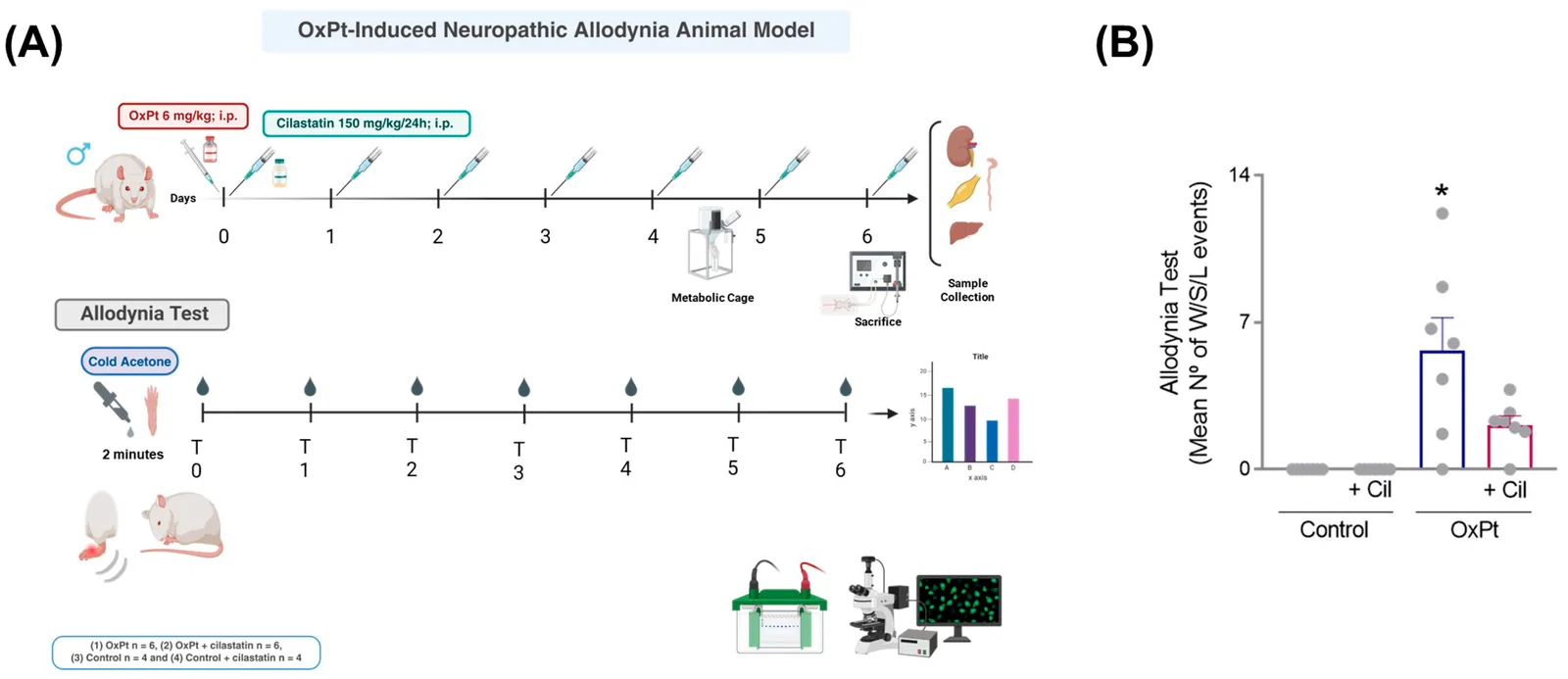
Experimental workflow and behavioral testing used for the study. (**A**) Assessment of thermal sensitivity using the acetone-induced cold allodynia test (T0–T6), performed by applying 200 μL of acetone onto the plantar surface of both hind paws. Nocifensive behaviors—including paw withdrawals, shakes, or licks—were recorded over a 2-min observation period. Reported values represent the mean of three trials conducted at 1-h intervals. (**B**) Cold allodynia assessment showing the median of three measurements of hind paw withdrawals (W), shakes (S), or licks (L) over the 6-day study period. All results are expressed as mean ± SEM; n = 4–6 animals per group. All data were analyzed using ANOVA. * *p* ≤ 0.0052 vs. all groups. OxPt: oxaliplatin; CIL: cilastatin.

**Figure 2:**
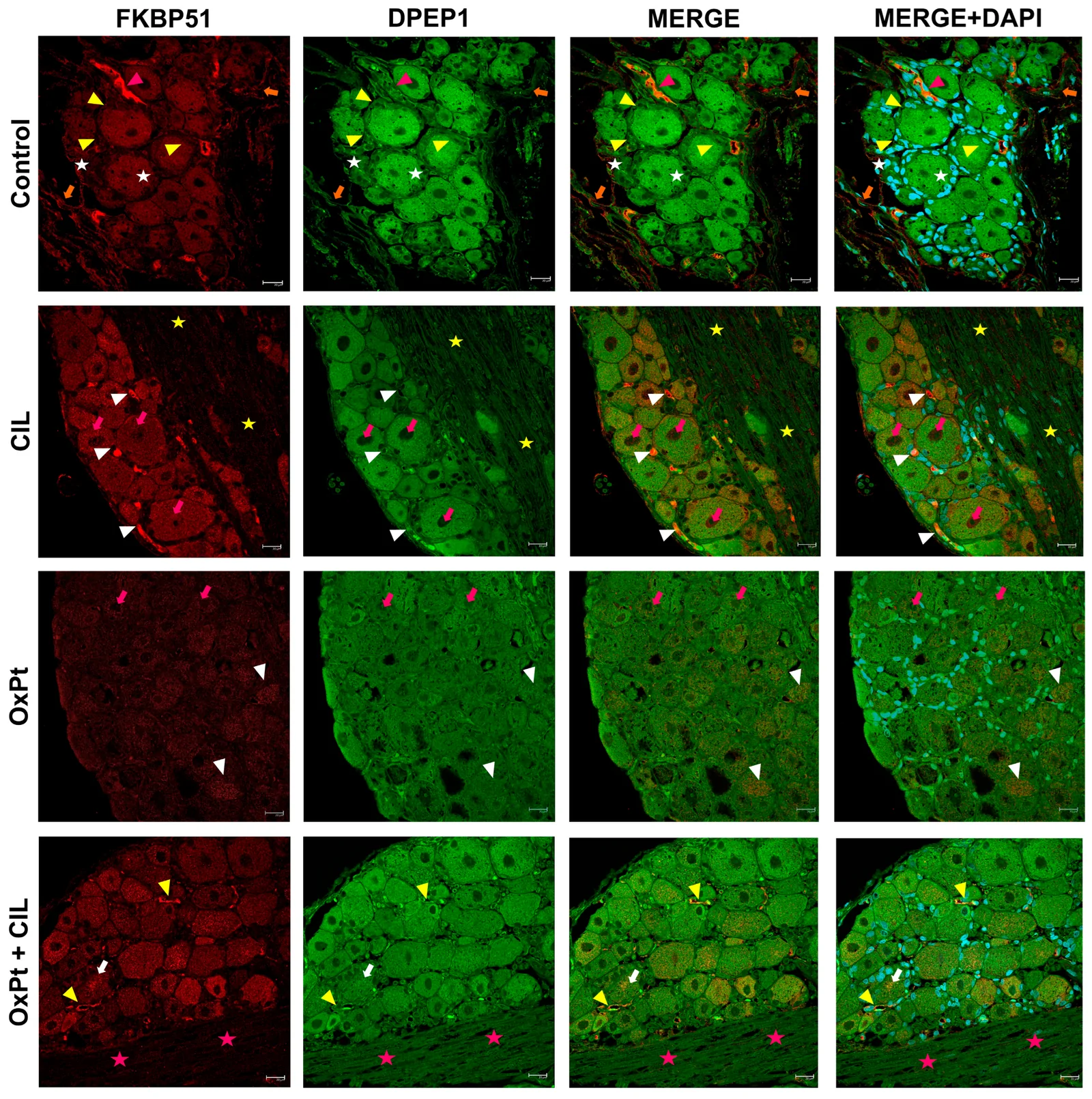
FKBP51 and DPEP1 co-expression in dorsal root ganglia. Immunostaining for FKBP51 (red) and DPEP1 (green). Control: FKBP51 appears at medium cytosolic dot-like intensity in neurons and glia (white stars). Strong colocalization is seen in neurons, the capsule (orange arrows), and endothelial/glial cells with the brightest FKBP51 (magenta arrowhead). DPEP1 shows high reticulated cytosolic signal in neurons and medium levels in satellite glial cells, separating both structures (yellow arrowheads). Cilastatin (CIL): FKBP51 appears brighter in endothelial and satellite glial cells (white arrowheads). Colabeling is evident in neurons, with DPEP1 predominant in cytosol and FKBP51 in nuclei. Spherical nuclear structures lacking FKBP51 but faintly positive for DPEP1 are present (magenta arrows). DPEP1 shows the same heterogeneous reticulated pattern as controls, with medium-to-high signal in axonal roots (yellow star). Oxaliplatin (OxPt): FKBP51 is mainly medium intensity in neurons and satellite glial cells, though some neurons display brighter dot-like cytosolic signals (white arrowheads). DPEP1 shows medium-to-high reticulated signal in neurons and glia, including cytosol and nuclei (magenta arrows). OxPt + CIL: FKBP51 is brighter in endothelial and satellite glial cells (yellow arrowheads), localized in cytosolic dots and neuronal nuclei (white arrow), with medium-to-low levels in roots (magenta star). Colocalization of both proteins is confirmed in merged images. DPEP1 appears at medium-to-high heterogeneous intensity in neurons, glia, and axonal roots. Scale bar, 20 μm.

**Figure 3:**
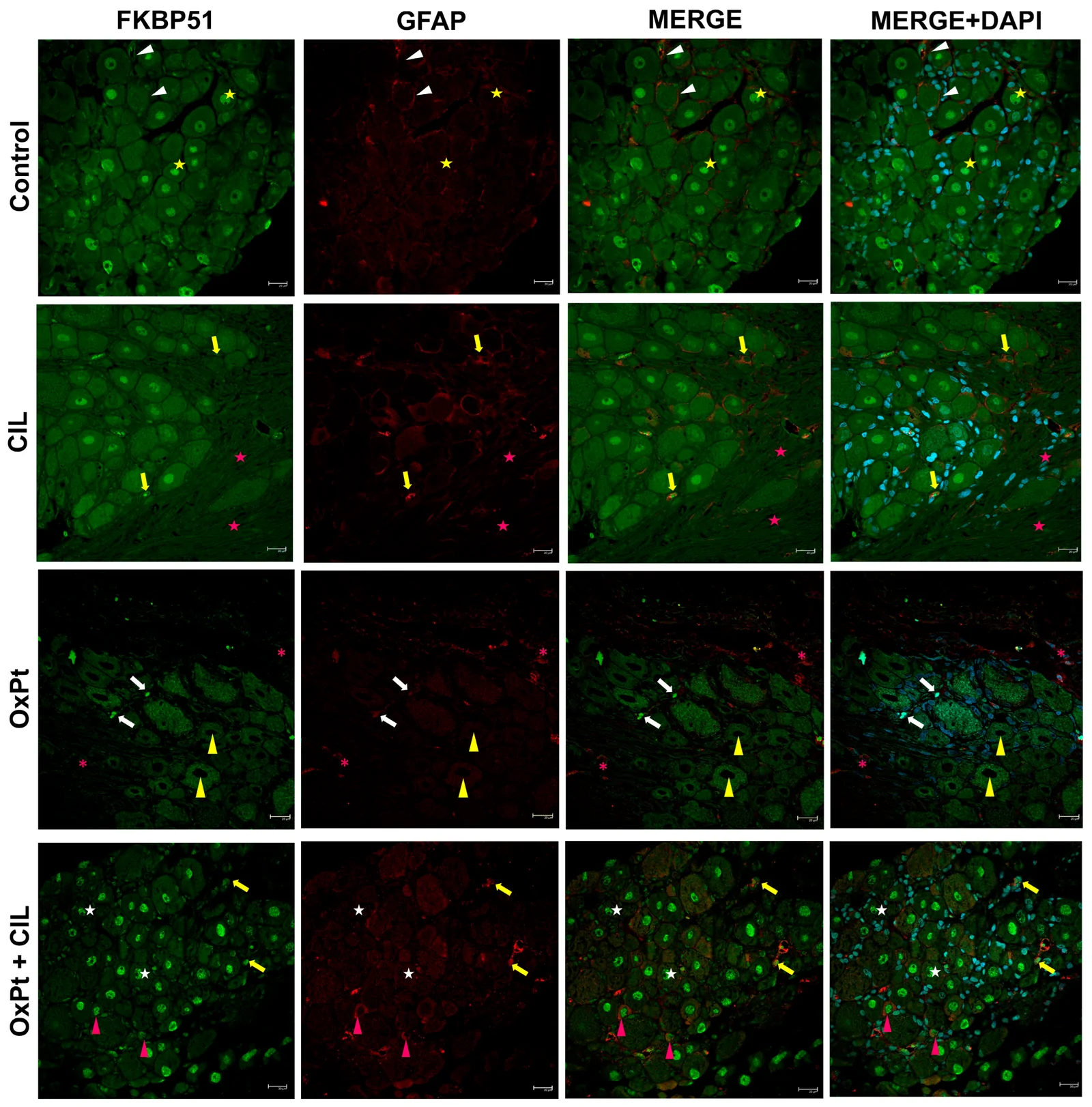
FKBP51 expression in glia. Immunostaining for FKBP51 (green) and GFAP (red). Control panels: FKBP51 signal at medium to high intensity in neurons. Fluorescence is present in several nuclei at the highest intensity (yellow stars). GFAP immunostaining in satellite glia cells (white arrowheads). FKBP51-GFAP co-immunostaining signal in glial cells. Cilastatin (CIL) panels: FKBP51 immunostaining shows a similar pattern and intensity to control panels, and a medium intensity signal in axons of the root (white stars). GFAP immunostaining signal in satellite glial cells at medium/high intensity and colocalizing with FKBP51 in some glia cells cytosol (yellow arrows). FKBP51-GFAP colocalization signal is present aside from axons in the root. Oxaliplatin (OxPt) panels: medium to high intensity FKBP51 signal in cytosol, in a reticulated pattern in some neurons (magenta stars), while in others there is a homogeneous dot pattern. Brightest FKBP51 immunostaining signal in some glia cell nuclei (white arrows). Faint or no signal for FKBP51 inside nuclei (yellow arrowheads). High-intensity GFAP signal in glial cells surrounding the axons of the root (magenta asterisks). Satellite glia cells around neurons depict GFAP signal (white arrows) along with light FKBP51-specific staining. OxPt + CIL panels: high-intensity immunofluorescence signal for FKBP51 in most neurons’ nuclei with absent signal in spherical spaces (hole-like) inside some of the nuclei (white stars). Medium/low intensity cytosolicFKBP51 signal in neurons in a homogeneous dot pattern. FKBP51 signal is also brighter inside nuclei than cytosol in glial cells (magenta arrowheads). GFAP immunofluorescence at variable intensity in satellite glial cells colocalizing with FKBP51 signal (yellow arrows). Scale bar, 20 μm.

**Figure 4:**
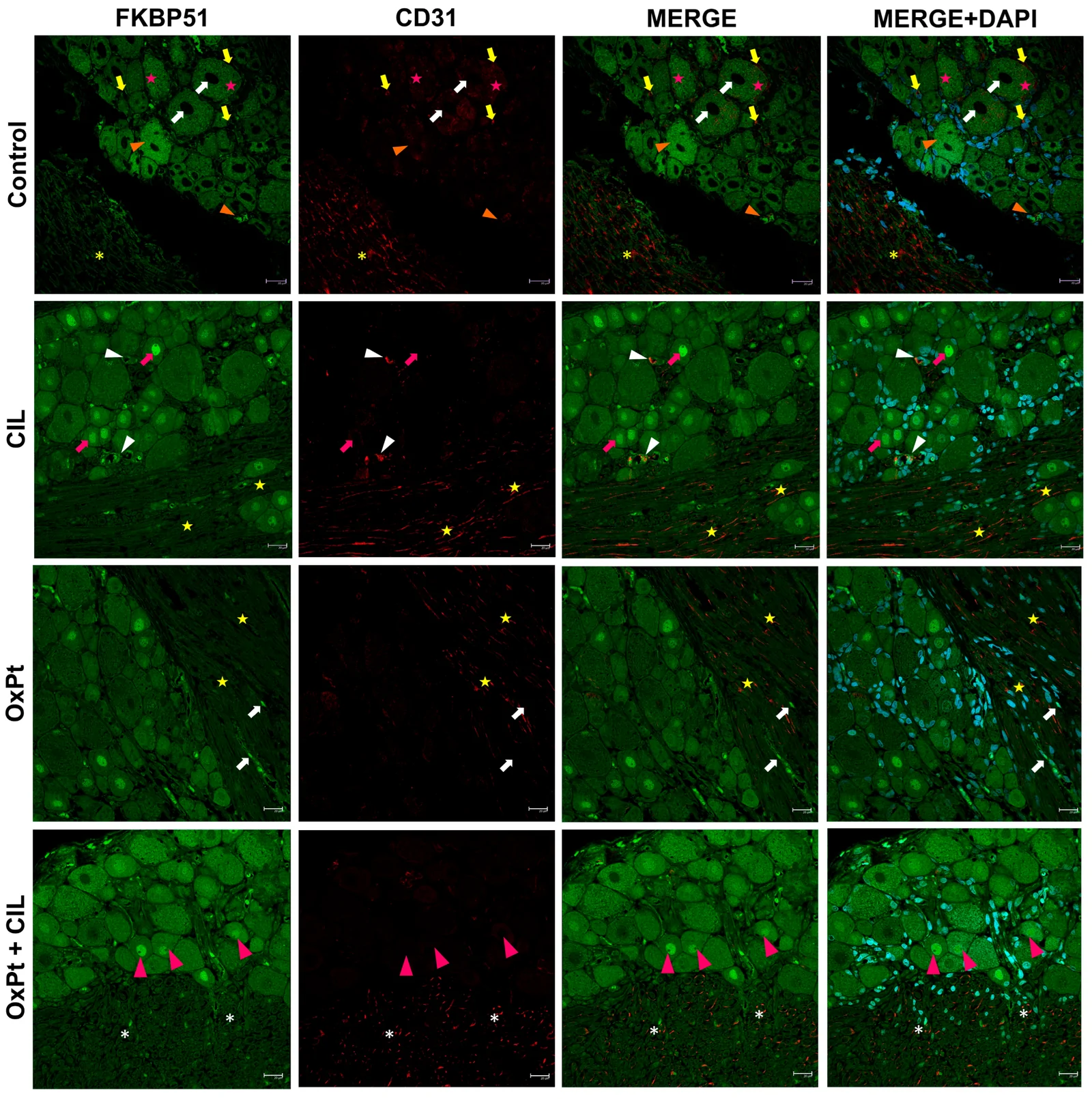
FKBP51 in blood vessels. Immunostaining for FKBP51 (green) and CD31 (red). Control panels: dot pattern of FKBP51 immunostaining signal in cells cytosol (magenta stars), faint signal in neurons and satellite glia cells nuclei (white arrows), and variable intensity in neurons/glia (orange arrowheads). CD31+ is present at a higher level in endothelial cells surrounding the root of axons (yellow asterisk) and endo- and perineurium. In the ganglion body, some points at medium intensity CD31 signal in endothelial cells around neurons (yellow arrows). Cilastatin (CIL) panels: FKBP51+ signal in heterogenous reticulated pattern in cytosol of neurons and glia cells. Brighter intensity in some nuclei of neurons and glia (magenta arrows). High level of CD31 in endothelial cells between axons of the root (yellow stars) and areas near neurons and glia bodies (white arrowheads) with evident co-labelling FKBP51-CD31 in merge. Oxaliplatin (OxPt) panels: FKBP51+ in a heterogeneous pattern in cytosol of neurons and glia at a medium intensity level with brighter intensity inside nuclei, including glia cells in axons of the root (white arrows). Medium/low intensity of CD31 signal in endothelial cells around axons in the root (yellow stars). OxPt + CIL panels: high-intensity signal for FKBP51 in a few glia and neuron cell nuclei, forming circle shapes with faint immunostaining signal (magenta arrowheads); endothelial cells show CD31 immunostaining in a network fashion in the root next to the ganglion (white asterisks). Scale bar, 20 μm.

**Figure 5:**
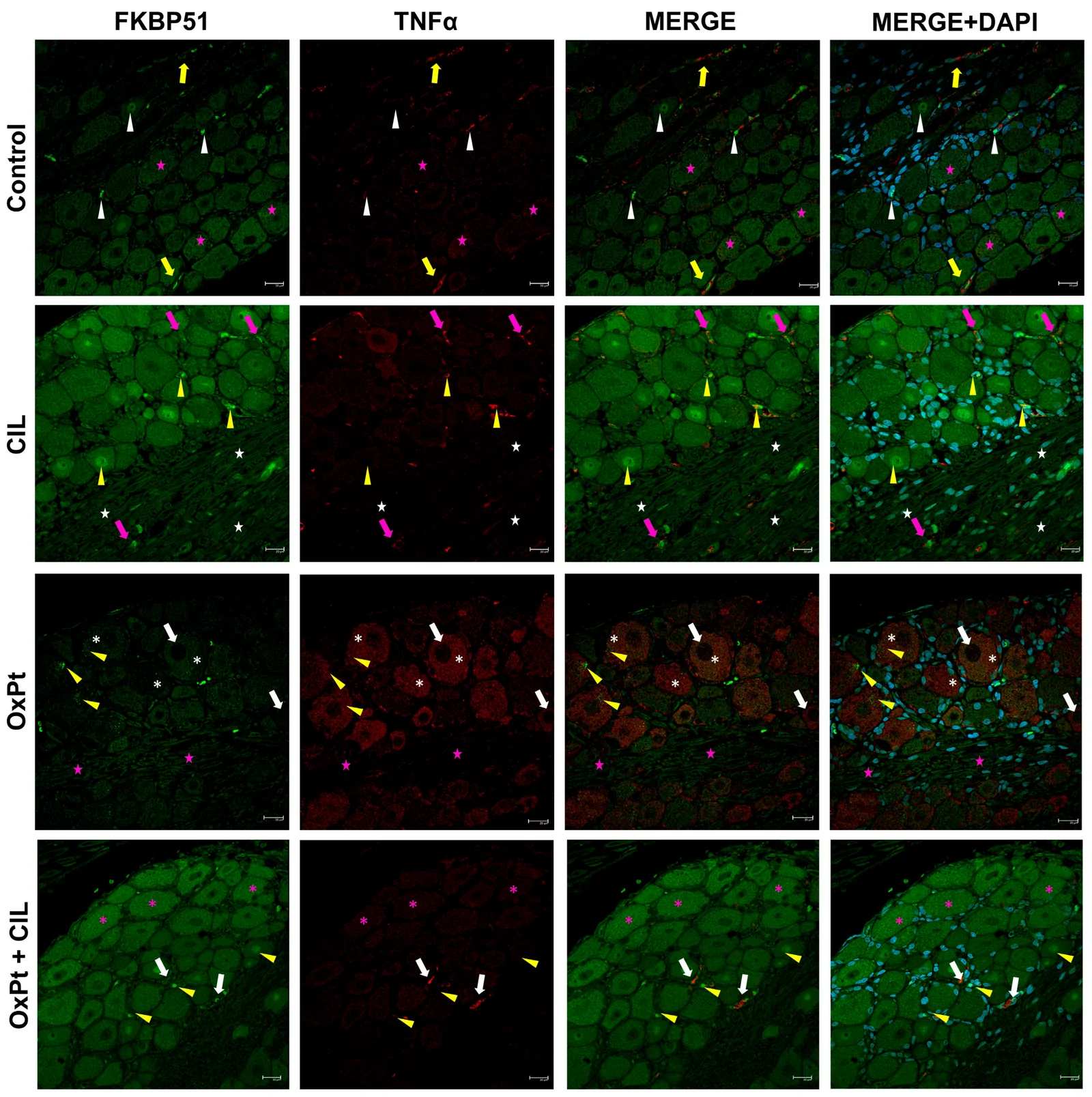
FKBP51 and TNFα expression. Control: FKBP51 (green) in neuronal cytosol (magenta stars) and nuclei (white arrowheads), TNFα (red) is low or absent (yellow arrows). Cilastatin (CIL): FKBP51, yellow arrowheads, and white stars (higher intensity in nuclei); TNFα in glia/endothelium (magenta arrows). Oxaliplatin (OxPt): lower fluorescence intensity for FKBP51, increased TNFα in neurons (white asterisk), with dot cytosolic pattern (white arrows), and low levels in axons (magenta stars). OxPt + CIL: nuclear FKBP51 fluorescence intensity restored (yellow arrowheads), TNFα in glia/endothelium (white arrows), absent in root. FKBP51 reticular pattern (magenta asterisks). Scale bar, 20 μm.

**Figure 6:**
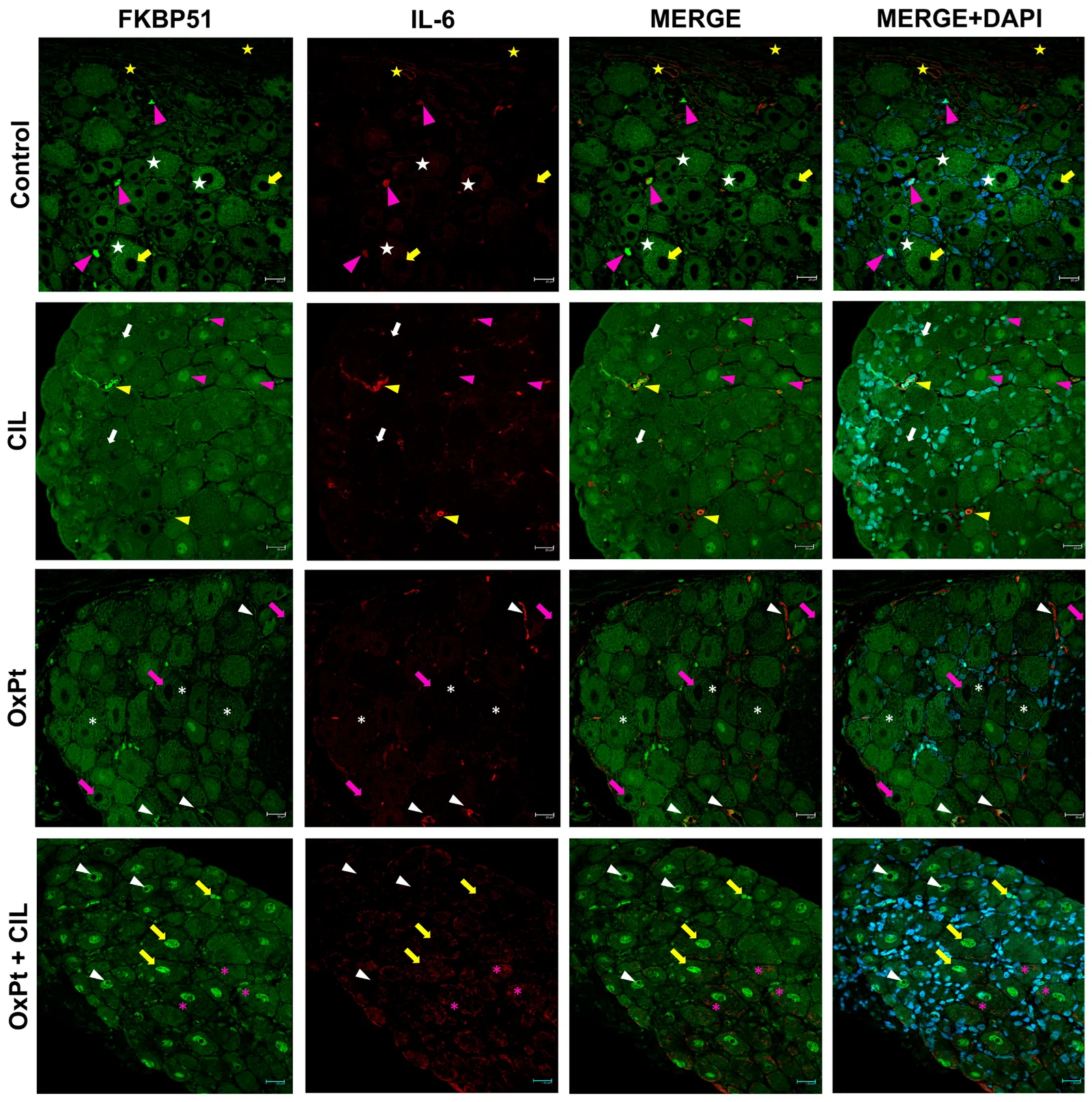
FKBP51 and IL-6 expression. FKBP51 (green) shows reticulated neuronal cytosolic signal (white stars), absent in most nuclei (yellow arrows), with weak IL-6 (red) in neurons and moderate in glia (magenta arrowheads and yellow stars). With cilastatin (CIL), FKBP51 increases in nuclei (magenta arrowheads) and cytosol (white arrows); IL-6 increases in glia/endothelium (yellow arrowheads). Oxaliplatin (OxPt) induces cytosolic FKBP51 (white asterisk) and weak nuclear signal (magenta arrows), with strong IL-6 in endothelium (white arrowheads). OxPt + CIL enhances nuclear FKBP51 (yellow arrows, white arrowheads) and shows moderate IL-6 in neurons (magenta asterisks) and faint in glia/endothelium. Scale bar, 20 μm.

**Figure 7:**
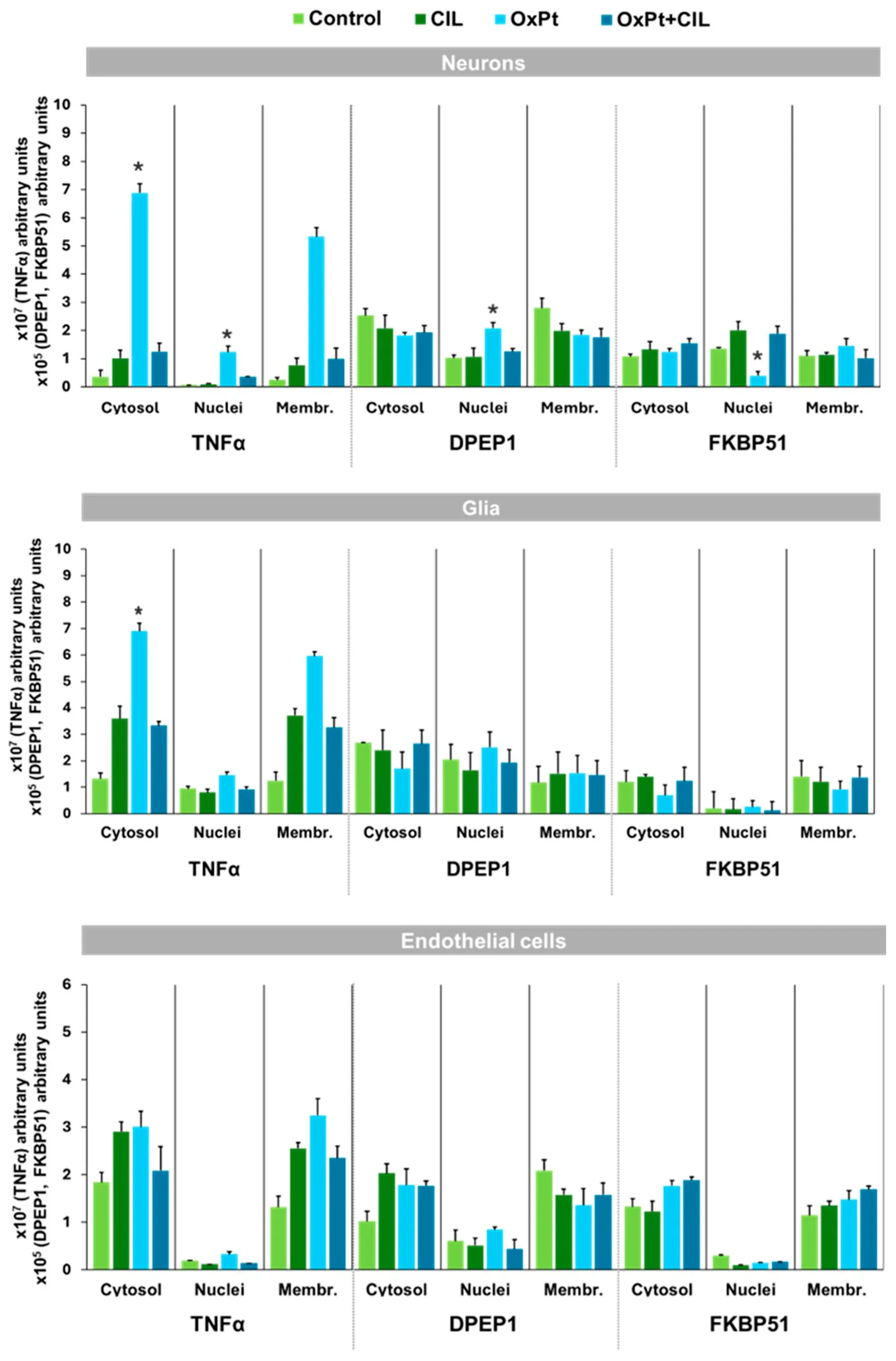
Intensity of specific fluorescence signal for TNF-α, DPEP1, and FKBP51 in DRG neurons in control, cilastatin (CIL), oxaliplatin (OxPt), and OxP + CIL treated groups of rats. TNF-*α* signal intensity was predominantly increased in the cytosol, nuclei, and membranes of neurons and cytosol of glial cells in OxPt-treated animals. The DPEP1 immunostaining signal was heterogeneous among the population of neurons, and increased signal was evident in nuclei of neurons in samples of OxPt-treated rats. The FKBP51 immunofluorescence signal showed heterogeneous pattern distribution, and decreased levels were observed in OxPt-treated animals, being significant in nuclei of neurons. Data are expressed as the mean ± SEM, * indicates statistical significance *p* < 0.05.

**Table 1:** Antibodies used in this study. * Color of fluorescence signal in confocal images.

Antibody	M/P	Dilution	Source	Cat.#	Figure (Fluorescence) *
Anti-DPEP1	Rabbit polyclonal	1:100	Martín-Vasallo/Ávila [17]	DPEP1C	2 (Green)
Anti-GFAP	Mouse monoclonal	1:100	Santa Cruz Biotechnology	sc-33673	3 (Red)
Anti-FKBP51	Rabbit polyclonal	1.25:100	Abcam	ab46002	3, 4, 5, 6 (Green)
Anti-FKBP51	Mouse monoclonal	1:100	Santa Cruz Biotechnology	sc-271547	2 (Red)
Anti-CD31	Mouse monoclonal	1:150	Santa Cruz Biotechnology	sc-376764	4 (Red)
Anti-TNFα	Mouse monoclonal	1:150	Santa Cruz Biotechnology	sc-52B83	5 (Red)
Anti-IL-6	Mouse monoclonal	1:200	Santa Cruz Biotechnology	sc-28343	6 (Red)
Anti-rabbit IgG (whole molecule), FITC-conjugated	Goat pAb against rabbit IgG	1:200	Sigma-Aldrich	F9887	2, 3, 4, 5, 6 (Green)
Anti-mouse IgG, DyLight^®^650-conjugated	Goat pAb against mouse IgG	1:100	Abcam	ab97018	2, 3, 4, 5, 6 (Red)
Anti-IL-6	Mouse monoclonal	1:200	Santa Cruz Biotechnology	sc-28343	6
Anti-rabbit IgG (whole molecule), FITC-conjugated	Goat pAb against rabbit IgG	1:200	Sigma-Aldrich	F9887	2, 3, 4, 5, 6
Anti-mouse IgG, DyLight^®^650-conjugated	Goat pAb against mouse IgG	1:100	Abcam	ab97018	2, 3, 4, 5, 6

**Table 2:** Allodynia test: mean number of hind paw withdrawals, shakings, or lickings over the 6-day duration of the model. All results are expressed as mean ± SEM; n = 4–6 animals per group. All data were analyzed using ANOVA.

Test	Control	CIL	OxPt	OxPt + CIL
Day 0	0	0	0	0
Day 1	0	0	1.67 ± 0.84	1.67 ± 0.80
Day 2	0	0	4.33 ± 2.19 #	2.67 ± 1.00
Day 3	0	0	6.67 ± 1.12 *	3.83 ± 0.75 **
Day 4	0	0	6.00 ± 1.00 *	2.33 ± 0.95
Day 5	0	0	12.17 ± 2.53 *	1.83 ± 0.40
Day 6	0	0	8.67 ± 2.65 *	2.33 ± 0.61

* *p* ≤ 0.0169 vs. all groups. # *p* ≤ 0.0578 vs. Control ± CIL; ** *p* ≤ 0.0053 vs. Control ± CIL. OxPt: oxaliplatin; CIL: cilastatin.

## Data Availability

The data presented in this study are available on request from corresponding authors.

## Supplementary Materials

The following supporting information can be downloaded at: https://www.mdpi.com/article/10.3390/cells15191737/s1, Supplementary Material S1: Table S1. Qualitative and semi-quantitative image analysis: specifying the intensity of specific fluorescence signal for DPEP1, FKBP51, GFAP, CD31, TNFα, and IL-6 in DRG cell types and subcellular localization (cytosol, nuclei and plasma membrane (p.membr.) from control, cilastatin (CIL) oxaliplatin-treated (OxPt) and OxPt + cilastatin treated (OxPt + CIL) groups of rats. Intensity levels: no staining (0), basal or faint (½), low (1), medium (2), and high (3). Figure S1: Example of specific fluorescence quantification for FKBP51 (red) and DPEP1 (green) in neurons in samples from the four groups (Control, cilastatin (CIL), oxaliplatin (OxPt), and OxPt + CIL) in cytosol and nuclei. Each lane shows both the red and green channels, the Regions of interest (ROIs) assigned to each cell (6–8 ROIs for large cells, 6–8 for intermediate cells, and 9–18 for small cells). For each photo, there are two slides: one showing the ROIs in the cytoplasm of each cell in the red and green channels, and the second showing the ROIs in the nucleus, which corresponds to the same ROI in the cytoplasm. For instance, if a cell has ROI number 6 in the cytoplasm, it will also have ROI 6 in its nucleus. This ensures that data number 6 in the spreadsheet (Excel or SPSS v29.0) corresponds to the same cell. Figure S2: Total glia was evaluated by measuring GFAP-specific fluorescence as a whole ROI in every quantified photography. CIL, cilastatin; OxPt, oxaliplatin. Figure S3: Specific fluorescence quantification examples for FKBP51 (red) and DPEP1 (green) in satellite glial cells in samples from the four groups (control, cilastatin (CIL), oxaliplatin (OxPt) and OxPt + CIL). Each lane shows both the red and green channels, the Regions of interest (ROIs), assigned to each cell, 30 measurements/ROIs per image. Figure S4: Total inflammation level was evaluated by measuring TNFα-specific fluorescence as a whole ROI in every quantified photography. CIL, cilastatin; OxPt, oxaliplatin. Figure S5: Colocalization analysis of DPEP1 (Ch1) and FKBP51 (Ch2) under different treatments. Representative scatterplots generated with FIJI (Colocalization Threshold) showing pixel-by-pixel intensity correlation between DPEP1 (Ch1) and FKBP51 (Ch2) in the four experimental groups (control, cilastatin (CIL), oxaliplatin (OxPt) and OxPt+ CIL). Colocalization analysis was performed using Colocalization Threshold plugin, applying Costes’ automatic thresholds for both channels. Each point represents a single pixel, and color scale indicates point density (from purple to yellow/white). Control cells display a characteristic high-intensity cluster with moderate dispersion. CIL alone maintains a similar pattern with slightly increased density. OxPt induces a marked compression of the point cloud, reducing intensity variability and producing a highly compact distribution. Co-treatment OxPt + CIL partially restores the dispersion pattern, indicating recovery of intensity heterogeneity. Pearson’s correlation coefficient above threshold and Manders’ overlap coefficients (M1, M2) were calculated, and statistical significance of colocalization was assessed using Costes’ randomization test. Figure S6: Differences due to the triple interaction (Treatment*Color*Size) on the average intensity. Cil, cilastatin; OxPt, oxaliplatin. Figure S7: Examples of Nuclear line intensity profile (NLIP). (A) NLIP comparison among neurons from the four study groups. (B) Images from Figure 2 of the manuscript and graph of NLIP obtained. CIL, cilastatin; OxPt, oxaliplatin. AF647-T1, DyLight^®^650; FITC-T2, Fluorescein IsoTioCyanate. Supplementary Material S2: Table S1: Numerical coding applied to the categories of the analyzed variables. CIL, cilastatin; OxPt, oxaliplatin. Table S2: Minimum and maximum area values used for intensity measurement in each treatment, along with their categorization according to the Size variable. Table S3: Recoding of the Size variable based on the measurement area. Table S4: Summary of the linear mixed model for FKBP51 fluorescence intensity (Mean Gray Value). Table S5: Summary of the linear mixed model for DPEP1 fluorescence intensity (Mean Gray Value).

## Abbreviations

The following abbreviations are used in this manuscript:

CD31	Cluster of differentiation 31
CDK11A	Cyclin Dependent kinase 11A
CDK15	Cyclin Dependent kinase 15
CDK4	Cyclin-dependent kinase 4
CDK9	Cyclin-dependent kinase 9
CDKL1	Cyclin-dependent kinase-like 1
CLK3	CDC Like kinase 3
CIL	Cilastatin
CIPN	Chemotherapy-induced peripheral neuropathy
CRC	Colorectal cancer
DPEP1	Dipeptidase 1
DRG	Dorsal root ganglia
FANCG	Fanconi anemia group G protein
FKBP51	FK506-binding protein 51
GFAP	Glial fibrillary acidic protein
GR	Glucocorticoid receptor
IKK	IkB kinase
IL-6	Interleukin 6
MCC	Manders’ coefficient
MOS	Proto-oncogene serine/threonine-protein kinase
NCAPD2	Non-SMC condensin I complex subunit D2
NCAPG	Non-SMC condensin I complex subunit G
NCAPH	Non-SMC condensin I complex subunit H
NF-κB	Nuclear factor kappa-light-chain-enhancer of activated B cells
OIPN	Oxaliplatin-induced peripheral neuropathy
ORC5	Origin recognition complex subunit 5
OxPt	Oxaliplatin
PCC	Pearson’s coefficient
PFA	Paraformaldehyde
PIPOX	Pipecolic acid and sarcosine oxidase
PPIase	Peptidyl-prolyl-(cis/trans)-isomerase
PSKH2	Protein serine/threonine-protein kinase H2
ROI	Region of interest
SMC2	Structural maintenance of chromosomes 2
SMC4	Structural maintenance of chromosomes 4
STK11	Serine/threonine kinase 11
SUN1	Sad1 and UNC-84 domain-containing protein 1
TNFα	Tumor necrosis factor alpha
ULK3	Unc-51 like kinase 3
VDAC1	Voltage-dependent anion-selective channel 1
WDR90	WD repeat domain 90
