# Supplementary material for: Cilastatin Protects Against Chemotherapy-Induced Neuropathy via the FKBP51/DPEP1 Axis: Confocal Insights into Protein Dynamics
Source: Cells. 2026 Sep 23;15(19):1737. doi: 10.3390/cells15191737 (PMC13663939; doi:10.3390/cells15191737)
Supplement: Supplementary file 1 [file cells-15-01737-s001.zip › Supplementary_Material_S1.pdf]

Supplementary Material File S1

Supplemental data regarding quantification of photography's-data and statistics analysis and modeling.

Table S1. Qualitative and semi-quantitative image analysis specifying the intensity of specific fluorescence signal for DPEP1, FKBP51, GFAP, CD31, TNFα and IL-6 in DRG cell types and subcellular localization (cytosol, nuclei and plasma membrane (p.membr.)) from control, cilastatin (CIL) oxaliplatin-treated (OxPt) and OxPt + CIL-treated (OxP t CIL) groups of rats. Intensity levels: no staining (0), basal or faint (½), low (1), medium (2), and high (3).

Cell type		**Neurons**			**Glia**			**Endothelial**		
Localization		Cytosol	Nuclei	Membr.	Cytosol	Nuclei	Membr.	Cytosol	Nuclei	Membr.
Marker	*Condition*									
FKBP51	*Control*	2/3	½	1/2	2/3	0	2/3	3	0	2/3
	CIL	2/3	2/3	2/3	2/3	0	1/2	3	0	2/3
	OxPt	1/2	½	1/2	1/2	0	1/2	2/3	0	1/2
	OxPt CIL	2/3	1/2	1/2	2/3	0	2/3	3	0	2/3
DPEP1	*Control*	2/3	1/2	2/3	1/2	2/3	2/3	1/2	3	1/2
	CIL	2/3	1/2	2/3	1/2	1/2	2/3	1/2	2/3	1/2
	OxPt	2/3	2	2	1/2	2/3	2/3	1/2	3	1/2
	OxPtCIL	2/3	1/2	2/3	1/2	1/2	2/3	1/2	3	2/3
GFAP	*Control*	0	0	0	1/2	0	1/2	0	0	0
	CIL	0	0	0	2/3	0	2/3	0	0	0
	OxPt	0	0	0	1/2	0	1/2	0	0	0
	OxPtCIL	0	0	0	2/3	0	2/3	0	0	0
CD31	*Control*	0	0	0	0	0	0	1/2	0	1/2
	CIL	0	0	0	0	0	0	1/2	0	1/2
	OxPt	0	0	0	0	0	0	2/3	0	2/3
	OxPtCIL	0	0	0	0	0	0	2/3	0	2/3
TNFα	*Control*	1	0	1	½/1	0	½	2/3	0	2/3
	CIL	1	0	1	½/1	0	½	2/3	0	2/3
	OxPt	2/3	½	2/3	1/2	0	1/2	2	0	2
	OxPtCIL	1	0	1	½/1	0	½	2/3	0	2/3
IL-6	*Control*	½	0	½	½	0	0	1/2	0	1/2
	CIL	½/1	0	½/1	1/2	0	1/2	1/2	0	1/2
	OxPt	½/1	0	½/1	½/1	0	½	1/2	0	1/2
	OxPtCIL	½/1	0	½/1	½/1	0	½	1/2	0	1/2

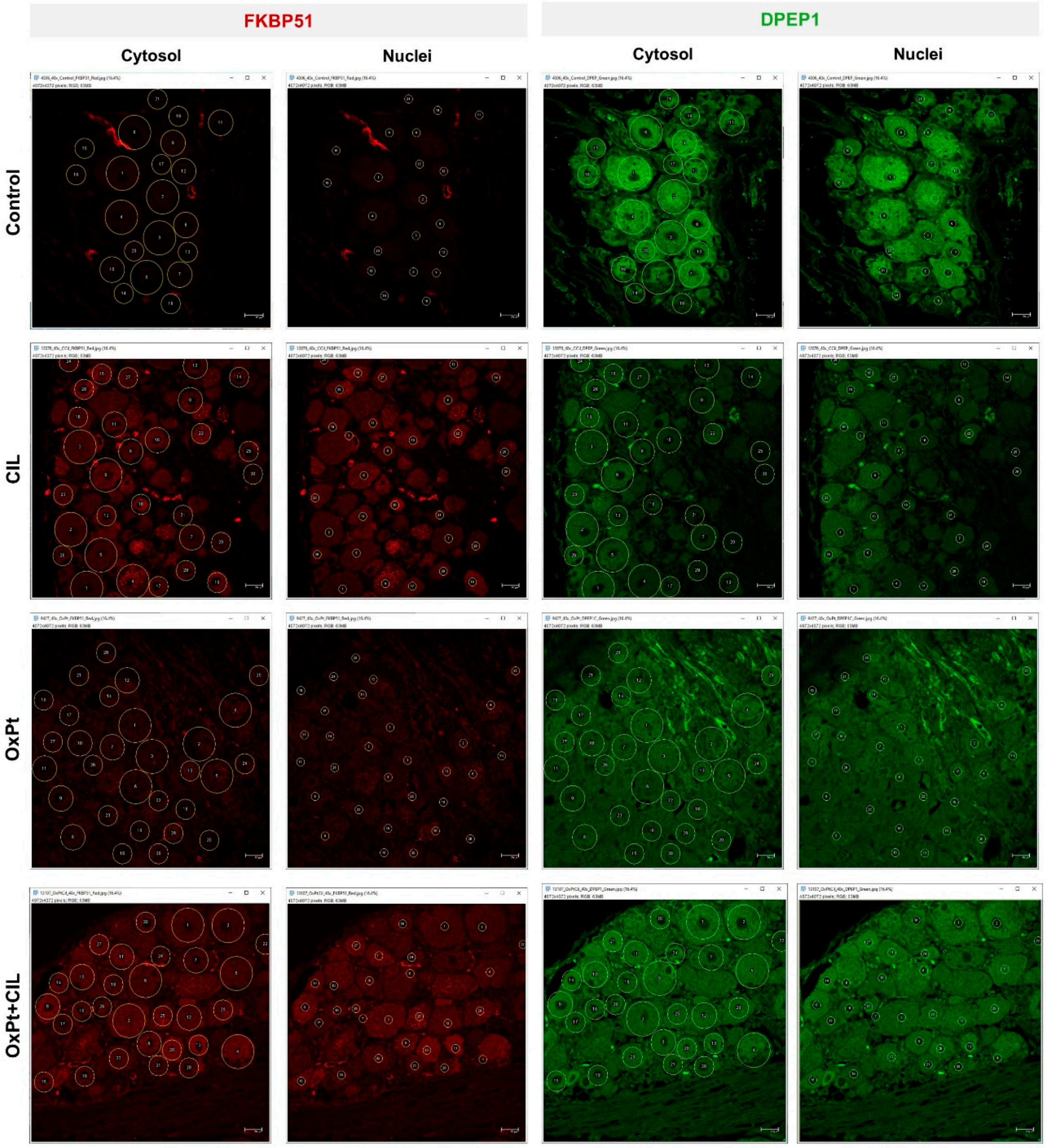

Figure S1. Example of specific fluorescence quantification for FKBP51 (red) and DPEP1 (green) in neurons in samples from the four groups (Control, cilastatin (CIL), oxaliplatin (OxPt) and OxPt + CIL) in cytosol and nuclei. Each lane shows both the red and green channels, the Regions of interest (ROIs) assigned to each cell (6-8 ROIs for large cells, 6-8 for intermediate cells, and 9-18 for small cells). For each photo, there are two slides: one showing the ROIs in the cytoplasm of each cell in the red and green channels, and the second showing the ROIs in the nucleus, which corresponds to the same ROI in the cytoplasm. For instance, if a cell has ROI number 6 in the cytoplasm, it will also have ROI 6 in its nucleus. This ensures that data number 6 in the spreadsheet (Excel or SPSS) corresponds to the same cell. Scale bar, 20 μm.

Corresponding Excel files were elaborated. There are two tabs, CYTOPLASM and NUCLEI. Both contain all the data generated by the Image J (Fiji) program using the corresponding ROIs.

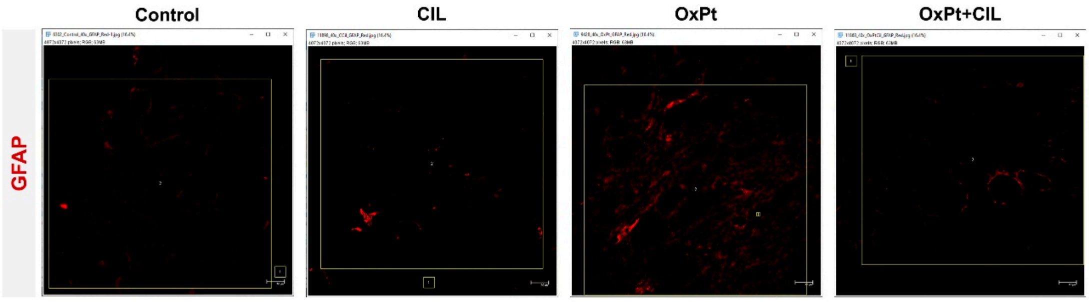

Figure S2. Total glia was evaluated by measuring GFAP-specific fluorescence as a whole ROI in every quantified photography. CIL, cilastatin; OxPt, oxaliplatin. Scale bar, 20 µm.

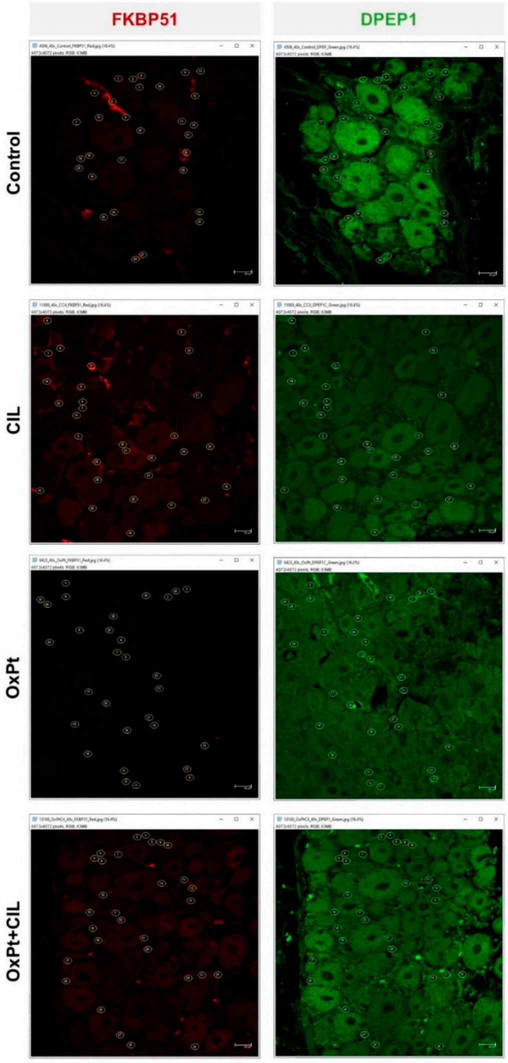

Figure S3. Specific fluorescence quantification examples for FKBP51 (red) and DPEP1 (green) in satellite glial cells in samples from the four groups (control, cilastatin (CIL), oxaliplatin (OxPt) and OxPt + CIL). Each lane shows both the red and green channels, the Regions of interest (ROIs), assigned to each cell, 30 measurements/ROIs per image. Scale bar, 20 µm.

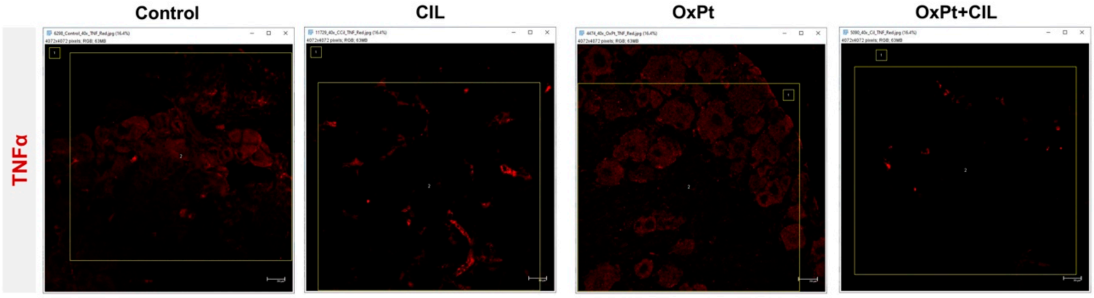

Figure S4. Total inflammation level was evaluated by measuring TNFα-specific fluorescence as a whole ROI in every quantified photography. CIL, cilastatin; OxPt, oxaliplatin. Scale bar, 20 μm.

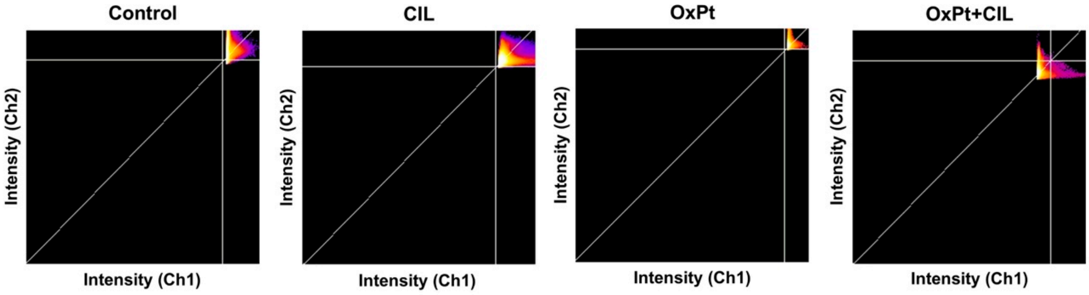

Figure S5. Colocalization analysis of DPEP1 (Ch1) and FKBP51 (Ch2) under different treatments. Representative scatterplots generated with FIJI (Colocalization Threshold) showing pixel-by-pixel intensity correlation between DPEP1 (Ch1) and FKBP51 (Ch2) in the four experimental groups (Control, cilastatin (CIL), oxaliplatin (OxPt) and OxPt + CIL). Colocalization analysis was performed using Colocalization Threshold plugin, applying Costes' automatic thresholds for both channels. Each point represents a single pixel, and color scale indicates point density (from purple to yellow/white). Control cells display a characteristic high-intensity cluster with moderate dispersion. CIL alone maintains a similar pattern with slightly increased density. OxPt induces a marked compression of the point cloud, reducing intensity variability and producing a highly compact distribution. Co-treatment OxPt + CIL partially restores the dispersion pattern, indicating recovery of intensity heterogeneity. Pearson's correlation coefficient above threshold and Manders' overlap coefficients (M1, M2) were calculated, and statistical significance of colocalization was assessed using Costes' randomization test.

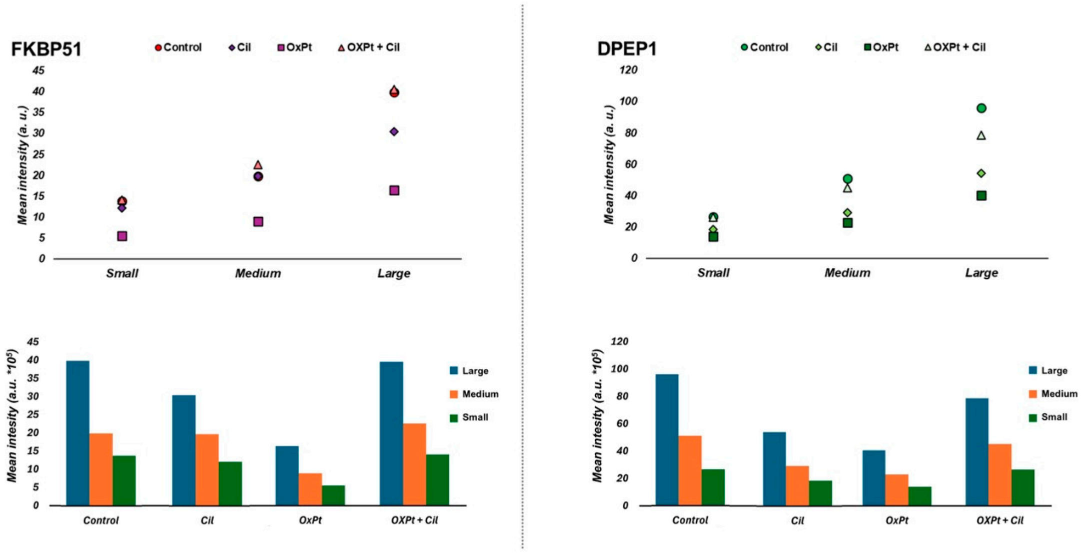

Figure S6. Differences due to the triple interaction (*Treatment*Color*Size*) on the average intensity. Cil, cilastatin; OxPt, oxaliplatin.

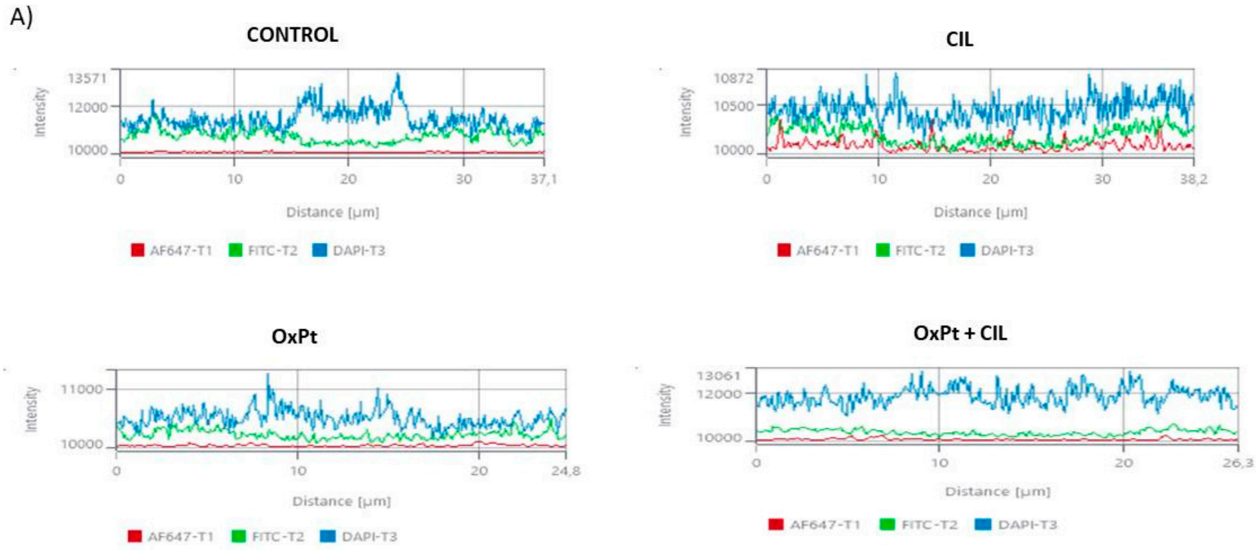

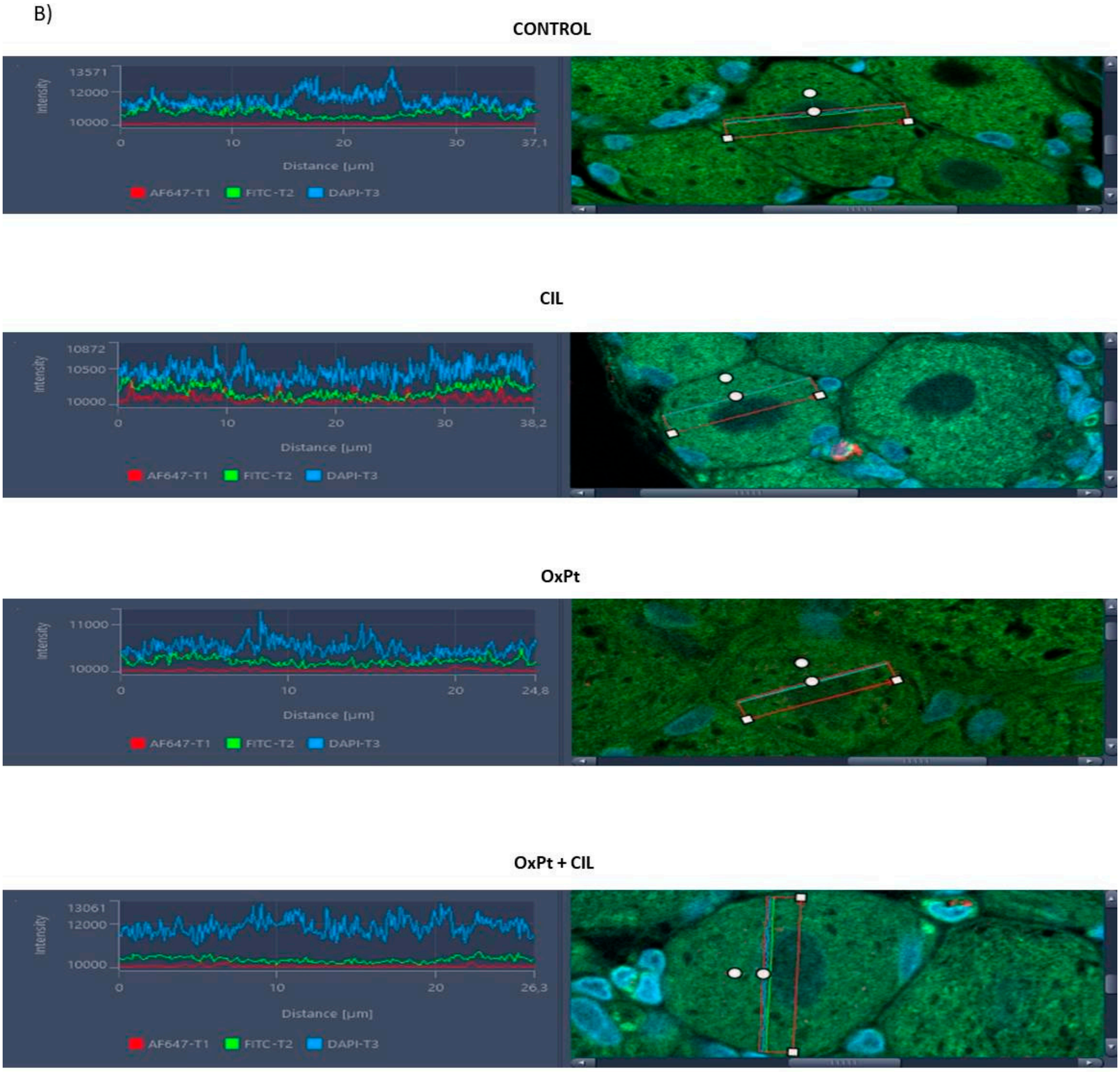

Figure S7. Examples of Nuclear line intensity profile (NLIP). A) NLIP comparison among neurons from the four study groups. B) Images from figure 2 of the manuscript and graph of NLIP obtained. CIL, cilastatin; OxPt, oxaliplatin. AF647-T1, DyLight®650; FITC-T2, Fluorescein IsoTioCyanate.
