# Supplementary material for: Cilastatin Protects Against Chemotherapy-Induced Neuropathy via the FKBP51/DPEP1 Axis: Confocal Insights into Protein Dynamics
Source: Cells. 2026 Sep 23;15(19):1737. doi: 10.3390/cells15191737 (PMC13663939; doi:10.3390/cells15191737)
Supplement: Supplementary file 1 [file cells-15-01737-s001.zip › Supplementary_Material_S2.pdf]

Supplementary Material File S2

For the statistics analysis of this study, the following aspects were considered:

1. The study was conducted in a homogeneous group of rats.

2. Four experimental conditions (treatments) were applied: control, T1, T2, and T3 (treatment variable).

3. For each treatment, dorsal root ganglia were dissected, immunocytochemistry was performed and confocal images were acquired.

4. For each image, two RGB channels were evaluated (color variable: Green and Red).

5. In each image and for each color channel, signal intensity was measured in three different types of areas corresponding to cells size (size variable: small [S], medium [M], and large [L]).

6. The number of measurements was not identical across size categories; however, each categories was measured more than once, but each type has always been measured more than once.

7. Thus, for each treatment, photo, channel, and size, the intensity value (continuous variable) is obtained.

The results were analyzed according to the dichotomous Variable *Position*: cytoplasm and nucleus. For each image, intensity measurement was obtained from the same cells using predefined regions of interest (ROIs).

For both cellular positions, sections were obtained and, in the corresponding confocal images, intensity signal were measured within a defined circular area. The Area variable collects the values corresponding to these measurement regions. The cells were classified into three groups based on the area size: large, medium, and small, across all images analyzed. This classification is represented by the variable *Size*.

The same measurement procedure was applied to two RGB channels, green and red defining the dichotomous variable *Color*.

For all images and treatments analyzed, ROIs were generally assigned as follows: ROIs 1-6 corresponded to large cells (six measurements), ROIs 7-12 to medium cells (six measurements), and ROIs 13-30 to small cells (18 measurements)

In the control group and related to cytoplasmatic measurement. In one image ROIs 1-7 corresponded to large cells (seven cells), ROIs 8-13 to medium cells (six cells), and ROIs 14-21 corresponded to small cells (eight cells). In the second image, the general scheme was maintained.

In the control group, nuclear measurements were obtained from a single image, with the following size distribution: ROIs 1 to 6 (six large cells), 7 to 12 (six medium cells), and 13 to 21 (nine small cells).

The numerical coding used is presented in Table S1.

Table S1. Numerical coding applied to the categories of the analyzed variables. CIl, cilastatin; OxPt, oxaliplatin.

Variable	*Category*	*Codification*
Position	cytoplasm	0
	nucleus	1
Treatment	control	0
	CIL	1
	OxPt	2
	OxPt + CIL	3
Size	small	0
	medium	1
	large	2
Color	DPEP1	0
	FKBP51	1

Table S2 presents the minimum and maximum area values used for intensity measurements for each treatment, along with the corresponding classification according to the size variable based on these values.

Table S2. Minimum and maximum area values used for intensity measurement in each treatment, along with their categorization according to the Size variable. CIL, cilastatin; OxPt, oxaliplatin

Treatment	*Size*	*Cases*	*Minimum*	*Maximum*
Control	small	52	91050	91050
	medium	24	91050	146872
	large	26	146872	258572
CIL	small	216	57954	91050
	medium	72	117510	146872
	large	72	209230	258572
OxPt	small	144	71884	91050
	medium	48	143756	146872
	large	48	141554	258572
OxPt + CIL	small	144	63110	91050
	medium	48	138478	146872
	large	48	256650	258572

Due to variability in the sizes of the areas measurement, and to ensure homogenous area categories across all treatments, the variable *Size* was recoded based on the area values presented in Table S3.

Table S3. Recoding of the Size variable based on the measurement area.

Size	*Cases*	*Minimum*	*Maximum*
Small	558	57954	95000
Medium	194	117510	146872
Large	190	209230	258572

Methods:

Fluorescence quantification was based on the ***Mean Gray Value*** (**Mean**) parameter exported from Fiji/ImageJ, which represents the average fluorescence intensity per pixel within each region of interest (ROI), independent of the cellular area.

The fluorescence channel (´Color´) was excluded as a fixed factor to avoid confounding technical variations (e.g., fluorophore properties, detector settings) with biological effects. Instead, FKBP51 and DPEP1 were analyzed independently. For each protein, a linear mixed model was established to evaluate the effects of Treatment (4 levels: Control, CIL, OxPt, Cil+OxPt) and Cell Size (3 levels: small, medium, and large, representing the relevant cellular compartments). The model included the main effects of both factors and their two-way interaction (Treatment x Cell Size).

To account for the nested structure of the data (multiple cells measured per image) and to control for technical baseline shift associated with the specific acquisition settings (including the fact that FKBP51 was predominantly detected in the red channel, but in the green channel in a minority of preparation), Image was included as a random effect. Post-hoc pairwise comparison with LSD adjustment were performed to explore significant interaction. Statistical significance was set at $p < 0.05$.

Results:

To evaluate the effect of the different treatments on **FKBP51** expression independently of the fluorescence channel, a linear mixed model was performed using the **Mean Gray Value** (**Mean**) as the dependent variable, with **Treatment** and **Cell Size** as fixed factors, and **Image** as a random effect. The analysis revealed a statistically significant **Treatment x Cell Size** interaction ($F(6, 447.03) = 2.900$, $p = 0.009$), indicating that the effect of the treatment on FKBP51 fluorescence intensity depends on the cellular size. Conversely the main effect of Treatment ($F(3, 12.05) = 2.595$, $p = 0.101$) and Cell Size ($F(2, 447.05) = 0.035$, $p = 0.966$) were not statistically significant.

Table S4. Summary of the linear mixed model for FKBP51 fluorescence intensity (Mean Gray Value)

Effect	*Intercept*	*Treatment*	*Cell Size*	*Treatment x Cell Size*
F – value	66.914	2.595	0.035	2.900
p – value	< 0.00001	0.101	0.966	0.009

Post-hoc analyses of the interaction revealed that the CIL treatment significantly altered FKBP51 fluorescence intensity specifically in small cells ($p = 0.031$), whereas no significant treatment effects were observed in medium or large cells ($p > 0.05$).

A linear mixed model was performed to evaluate the effect of the treatments on DPEP1 expression, using the Mean Gray Value as the dependent variable, with Treatment and Cell Size as fixed factors, and Image as a random effect.

The analysis revealed significant main effects for both Treatment ($F(3, 11.16) = 8.250$, $p = 0.004$) and Cell Size ($F(2, 418.17) = 6.370$, $p = 0.002$). Importantly, the Treatment x Cell Size interaction was also statistically significant ($F(6, 418.12) = 2.253$, $p = 0.038$), indicating that the effect of the treatment on DPEP1 fluorescence intensity is modulated by the cellular size.

Table S5. Summary of the linear mixed model for DPEP1 fluorescence intensity (Mean Gray Value)

Effect	*Intercept*	*Treatment*	*Cell Size*	*Treatment x Cell Size*
F – value	288.81	8.250	6.370	2.253
p – value	< 0.00001	0.004	0.002	0.038

Post-hoc pairwise comparisons against the **Control** group revealed that both the **CIL** and **OxPt** treatments significantly decreased DPEP1 fluorescence intensity across all cell sizes (small, medium and large; all $p < 0.05$). In contrast, the combined **OxPt+CIL** treatment did not significantly alter DPEP1 levels compared to the **Control** in any cell size category (all $p > 0.05$).
